# Heterogeneous Brain Atrophy Sites in Anxiety Disorders Map to a Common Brain Network

**DOI:** 10.1155/2024/3827870

**Published:** 2024-04-02

**Authors:** Yinian Yang, Wenqiang Xu, Yingru Wang, Hai Cao, Xiaoqing Yao, Ting Zhang, Xiaohui Xie, Qiang Hua, Wen Cheng, Longshan Shen, Kongliang He, Yanghua Tian, Kai Wang, Gong-Jun Ji

**Affiliations:** ^1^Department of Neurology, The Second Affiliated Hospital of Anhui Medical University, Anhui Medical University, Hefei, China; ^2^School of Mental Health and Psychological Sciences, Anhui Medical University, 81 Meishan Road, Hefei 230032, China; ^3^Anhui Province Key Laboratory of Cognition and Neuropsychiatric Disorders, Hefei, China; ^4^Collaborative Innovation Centre of Neuropsychiatric Disorder and Mental Health, Hefei, China; ^5^Department of Neurology, The First Affiliated Hospital of Anhui Medical University, Anhui Medical University, Hefei, China; ^6^Key Laboratory of Computational Medicine and Intelligent Health of Anhui Higher Education Institutes, Bengbu Medical College, Bengbu, China; ^7^Bengbu Hospital of Shanghai General Hospital, China; ^8^The Second Affiliated Hospital of Bengbu Medical University, China; ^9^Anhui Mental Health Center, Hefei, China; ^10^Department of Psychology and Sleep Medicine, The Second Affiliated Hospital of Anhui Medical University, Anhui Medical University, Hefei, China; ^11^Hefei Comprehensive National Science Center, Institute of Artificial Intelligence, Hefei, China; ^12^Anhui Institute of Translational Medicine, Hefei, China

## Abstract

**Background:**

Heterogeneous findings among anxiety disorder studies have hindered elucidation of the underlying pathophysiology and the development of mechanism-based therapies.

**Purpose:**

To determine whether structural MRI findings in anxiety disorder studies converge on a common network with therapeutic significance.

**Materials and Methods:**

In this retrospective study, a systematic literature search of PubMed and Web of Science databases was performed to identify coordinates of gray matter atrophy in patients with anxiety disorder. Atrophy coordinates were then mapped to an anxiety network constructed from the resting-state functional MRI (rs-fMRI) data of 652 healthy participants using “coordinate network mapping” and validated by specificity tests. The causal association of this network to anxiety symptoms was tested in a cohort of patients with brain lesions and emergent anxiety symptoms. The potential therapeutic utility of this anxiety network was then assessed by examining the clinical efficacy of network-targeted repetitive transcranial magnetic stimulation (rTMS) among a separate anxiety disorder cohort. Statistical analyses of images were performed using nonparametric tests and corrected for family-wise error.

**Results:**

Sixteen studies comprising 453 patients with anxiety (245 females; mean age ± [SD], 31.4 ± 8.71 years) and 460 healthy controls (238 females; 31.7 ± 10.08 years) were included in the analysis. Atrophy coordinates were mapped to an anxiety network with a hub region situated primarily within the superficial amygdala. Lesions associated with emergent anxiety symptoms exhibited stronger connectivity within this anxiety network than lesions not associated with anxiety (*t* = 2.99; *P* = .004). Moreover, the connectivity strength of rTMS targets in the anxiety network was correlated with the improvements of anxiety symptom after treatment (*r* = .42, *P* = .02).

**Conclusions:**

Heterogeneous gray matter atrophy among patients with anxiety disorder localize to a common network that may serve as an effective therapeutic target.

## 1. Introduction

Anxiety disorders are among the most common psychiatric illnesses [1], afflicting up to 15% of the general population worldwide [2]. Negative emotions such as sadness and excessive worry are common manifestations of anxiety disorders, and the severity of these symptoms can fluctuate widely, interfering with daily function and reducing quality of life. Over the past few decades, numerous studies have identified structural brain abnormalities associated with anxiety disorders [3].

Voxel-based morphometry (VBM), one of the main structural MRI methods [4], has revealed heterogeneous atrophy locations across studies of anxiety disorders. Meta-analyses can help to resolve mixed findings but have failed in some critical regions, such as the amygdala. While the amygdala is an essential region regulating cognitive and behavioral aspects of anxiety [5–7], observed structural changes have differed across meta-analyses [3, 8, 9]. The heterogeneous findings between studies or meta-analyses have made it challenging to determine the brain network of anxiety, thereby hindering the exploration of mechanism-based clinical therapy, such as transcranial magnetic stimulation (TMS). One of the urgent requirements for repetitive transcranial magnetic stimulation (rTMS) treating anxiety is the localization of stimulation target. DLPFC may be an effective target for anxiety treatment [10, 11], but how to identify the optimized subregion of DLPFC is largely unknown. As illustrated in network-guided rTMS studies of depression [12–14], we predicted that the importance of DLPFC target in the anxiety network may be associated with clinical outcome.

Darby et al. [15] proposed a novel concept to reconcile these inconsistencies. In contrast to traditional assumptions that disease-associated abnormalities should be consistently localized to specific brain regions [16, 17], they proposed that brain abnormalities associated with neuropsychiatric diseases are more likely localized within common brain networks [18, 19]. This theory has been validated in a variety of neurology and psychiatry diseases, such as migraine [20], major depressive disorder [21], and transdiagnostic psychiatric disorders [22], using “coordinate network mapping” (CNM), a validated method derived from lesion network mapping [23] in which the coordinates of abnormalities from neuroimaging studies are mapped to independently defined networks [15]. While standard CNM alone cannot produce causal inferences, integration of CNM maps with maps derived from definitive causal associations, such as between localized brain lesions and emergent behavioral symptoms, may help establish causal relationships between brain abnormalities within networks and psychiatric disorders [24].

In this study, we examined if heterogeneously localized structural abnormalities revealed by VBM analyses of anxiety disorders map to a common brain network using CNM, and if lesions associated with emergent anxiety symptoms (but not unassociated lesions) also map to this network. Finally, we explored the clinical significance of this shared network by examining associations with symptom outcomes among patients receiving rTMS.

## 2. Materials and Methods

This retrospective study was approved by the institutional review board, and study procedures were performed in accordance with the ethical principles for medical research involving human subjects defined in the Declaration of Helsinki.

### 2.1. Study Sample

**Figure 1:**
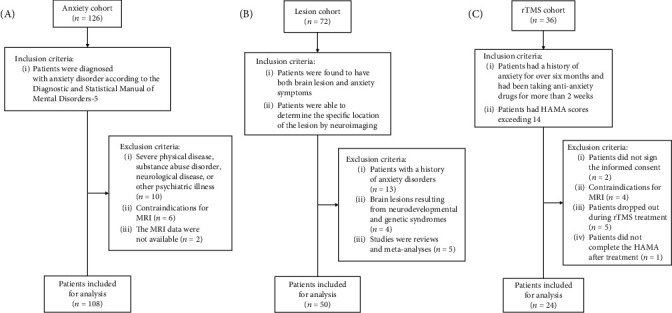
Flowcharts of the patient recruitment process. (A) Anxiety cohort; (B) lesion cohort; and (C) rTMS cohort. rTMS = repetitive transcranial magnetic stimulation, HAMA = Hamilton Anxiety Scale.

We searched PubMed and Web of Science databases from inception to November 8, 2022, for articles reporting gray matter volume reduction (atrophy) in anxiety disorder patients. The search string included terms related to or describing anxiety and VBM. Inclusion criteria and detailed search strategies are described in Supplementary S1 and Table S1. We also collected data from three cohorts of patients (inclusion and exclusion criteria shown in Figure 1): 108 patients diagnosed with anxiety disorders (anxiety cohort, 12 August 2020 to 8 August 2023), 50 patients with brain lesions (lesion cohort, 6 November 2019 to 21 September 2022), and 24 patients with anxiety symptoms who received rTMS (rTMS cohort, 26 September 2020 to 13 November 2022). In addition, we utilized a cohort of healthy participants (*n* = 652) from previous work (healthy cohort, demographic information is presented in Table 1) [25]. Written informed consent was obtained from the healthy, anxiety, and rTMS cohorts, whereas consent was not required from lesion cohort patients because all data were acquired during routine care.

### 2.2. Coordinate Network Mapping

Coordinate network mapping was performed as described in previous studies [15]. First, we extracted peak coordinates for brain atrophy reported in studies (*n* = 16) meeting the inclusion criteria. Any coordinates reported in Talairach space were converted to MNI space. Next, a 3 mm radius sphere centered on each coordinate was created for every study (larger and smaller spheres were also used for robustness testing, see Supplementary Figure 2 for 1.5 mm and 4.5 mm spheres). Multiple spheres extracted from a single study were combined to create a study-specific seed. Subsequently, resting-state functional connectivity between the study-specific seed and all other brain voxels was computed from the resting-state fMRI data of 652 healthy subjects using Pearson's correlations. These analyses were then repeated for the 108 patients of the anxiety cohort. Detailed scan sequences of this healthy connectome, together with data processing information, can be found in Supplementary S2. We performed a voxel-wise one-sample *t*-test on the 652 functional connectivity maps (Fisher's *z*-transformed) to obtain a study-specific network. These procedures were repeated for each study-specific seed. Finally, the 16 study-specific networks were binarized at *t* > 5.1 (corresponding to a voxel-wise family-wise error (FWE) rate of *P*_FWE_ < .01, see Supplementary Figure 2 for results of reanalysis using thresholds *t* > 7 and *t* > 9) and overlapped to define the common connectivity among all studies (termed the “sensitivity map”).

To identify regions in the “sensitivity map” also specific to anxiety, we compared the connectivity maps of anxiety coordinates with random maps and maps of nonanxiety disorders independently (see details in Supplementary S3), yielding a “random-controlled specificity map” and “disorder-controlled specificity map”, respectively.

### 2.3. Defining a Human Anxiety Network

To identify the hub region of anxiety disorders as in previous work [26], we performed a conjunction analysis between the highly overlapping regions of the sensitivity map (≥80% [13 of 16] or 75% [12 of 16] for the analysis in Supplementary Figure 2) and voxels surviving both specificity tests (random- and disorder-controlled maps). We defined the whole brain connectivity *t* map of the hub region as the “human anxiety network”, which was computed by the same methods for computing the study-specific *t* maps. Briefly, the resting-state functional connections between the hub region and other brain voxels were calculated using the resting-state fMRI data of 652 healthy subjects. Then, a voxel-wise one-sample *t*-test was performed on the 652 functional connectivity maps (Fisher's *z*-transformed) to obtain the “human anxiety network”.

### 2.4. Anxiety-Related Lesions

To test whether the anxiety network derived from cross-sectional studies overlaps with the locations of brain lesions causing anxiety symptoms, we enrolled a cohort of patients with brain lesions (some identified from the literature search, see details in Supplementary S4 and Table S2) and established two subgroups, anxiety lesion and nonanxiety lesion, according to the appearance of anxiety symptoms after lesion occurrence. All lesion masks were traced onto a standardized brain atlas (MNI152 T1 template with 2 mm resolution) using 3D Slicer software as described previously [27, 28]. The average *t-*value within each lesion was extracted from the anxiety network (a *t* map) and compared between the anxiety-lesion and nonanxiety-lesion groups using a two-tailed two-sample *t*-test.

### 2.5. Relevance of the Anxiety Network for Treatment Response

To examine if the defined network is an effective target for clinical treatment, 24 patients with anxiety symptoms who received 14 days of rTMS were analyzed retrospectively. All patients completed the HAMA assessments before and after treatment. Structural MRI scans were also acquired before rTMS for neuronavigation (detailed rTMS protocol in Supplementary S5).

We generated a 6 mm radius sphere centered on each patient's target coordinate and extracted the average value of this sphere region in the anxiety network. Since the anxiety network is a *t* map, we obtained a *t*-value for each patient's target. The *t*-value reflects the functional connectivity strength between the target and the hub region. We then conducted a correlation analysis (one tailed) to test whether the *t*-value predicted anxiety symptom improvement (improvement rate = [pretreatment HAMA score − posttreatment HAMA score]/pretreatment HAMA score). Correlation analysis was repeated with sex, age, years of education, and duration of disease included as covariates. Analysis was also repeated using a sphere radius of 3 mm.

### 2.6. Statistical Analysis

Image-based statistical analyses were performed using the SnPM tool in SPM12 (http://warwick.ac.uk/snpm), while all other analyses were conducted using SPSS 23 (https://www.ibm.com/spss). A *P* value of < .05 after correction for multiple comparisons was considered statistically significant for all tests. Lesion masks were traced using 3D Slicer software (https://www.slicer.org/). Additional details on analyses are provided in the relevant Methods sections.

## 3. Results and Discussion

### 3.1. Patient Characteristics

A literature search identified 16 studies of anxiety patients reporting gray matter atrophy in a total of 58 locations (coordinates). These studies included 453 patients with anxiety (245 females; mean age ± standard deviation, 31.37 ± 8.71 years) and 460 healthy controls (238 females; 31.7 ± 10.08 years) (see details in Supplementary Figure 1 and Table S3). Furthermore, we recruited three cohorts of patients (Table 1 and Figure 1): (1) an anxiety cohort of 108 anxiety disorder patients used to test the robustness of the anxiety network constructed from healthy controls, (2) a lesion cohort of 50 lesion patients for testing the causal relationship between the defined anxiety network and anxiety symptoms, and (3) a rTMS cohort of 24 patients with anxiety symptoms receiving rTMS to demonstrate the clinical significance of the anxiety network.

### 3.2. Mapping Heterogenous Atrophy Coordinates to a Common Brain Network

**Figure 2:**
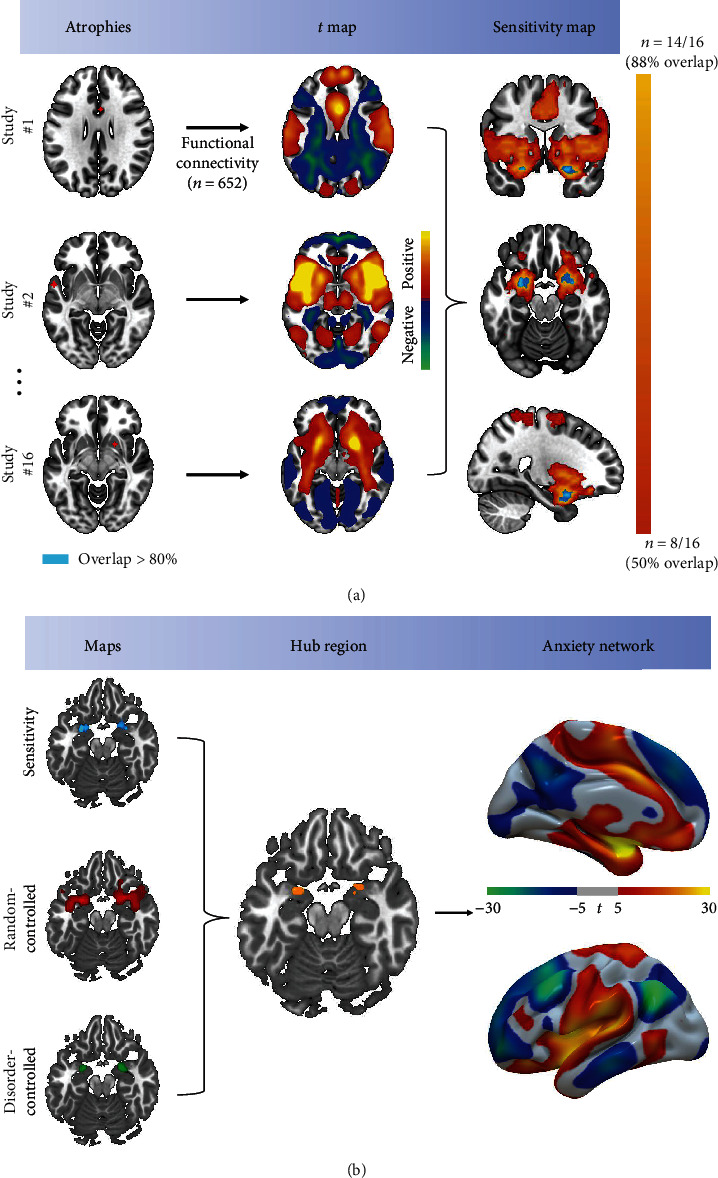
Gray matter atrophy sites in anxiety disorders map to a common neural network. The hub of this network was identified through sensitivity, specificity, and conjunction analysis. (a) Atrophy coordinates from each study (*n* = 16) were combined into a single study-specific seed. A whole-brain connectivity map of a given seed was computed using the rs-fMRI data of 652 healthy subjects and a one-sample *t*-test. The *t* maps for each study were thresholded, binarized, and overlapped to identify regions of shared connectivity (the “sensitivity map”). (b) Next, *t* maps of anxiety coordinates were compared with random connectivity maps and connectivity maps of nonanxiety disorders, yielding two specificity maps (random controlled and disorder controlled). The conjunction of our sensitivity (≥80%, 13 of 16) and specificity analyses identified a hub region in the bilateral amygdala. The connectivity pattern of the hub region across 652 healthy subjects is termed the anxiety network.

The locations of atrophy were highly heterogeneous across the 16 studies (Figure 2(a)). To reconcile this spatial heterogeneity with the shared symptoms of anxiety, we tested whether these atrophy sites (coordinates) could be mapped to a common connected brain network using coordinate network mapping. The resulting “sensitivity map” showed that 14 of 16 study-level coordinates (88%) were functionally connected to the left amygdala (Figure 2(a)). The robustness of this sensitivity map was tested by repeating the analysis using another independent resting-state fMRI dataset of anxiety patients, using each coordinate as an individual seed, using two higher *t*-value thresholds defining study-specific networks, and using two additional seed sizes (Supplementary Figure 2). In the specificity analysis, the networks of 16 study-level coordinates showed higher functional connectivity as compared to the networks of random coordinates and networks of nonanxiety disorders (*n* = 85, see details in Table S4 and S5) (Figure 2(b), *P*_FWE_ < .05).

Conjunction analysis identified a hub region primarily situated within the bilateral superficial amygdala [29] (SFA, peak MNI coordinates: [-21 3-21], [24 6-21]) showing both sensitivity (>80%) and specificity (*P*_FWE_ < .05) for anxiety disorders. We defined the whole-brain functional connectivity map of this hub region as the anxiety network (Figure 2(b)). This network pattern was independent of sensitivity map threshold (see the 75% threshold result in Supplementary Figure 3).

### 3.3. Causal Implication of the Network to Anxiety Symptoms

**Figure 3:**
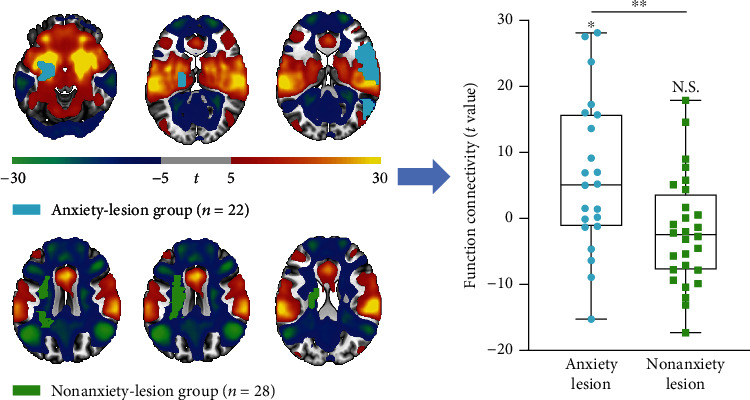
Demonstration of a causal relationship between the common network and anxiety symptoms. Lesions in the anxiety group showed significant (one-sample *t*-test *t* = 2.58, *P* = .02) and stronger functional connectivity (two-sample *t*-test *t* = 2.99, *P* = .004) within the anxiety network than lesions not associated with anxiety. ⁣^∗^*P* < .05, ⁣^∗∗^*P* < .01, N.S. = not significant.

We collected lesion data from 22 patients with anxiety symptoms and 28 without anxiety symptoms (17 patients were identified from literature search, see flowchart in Supplementary Figure 4). These groups did not differ in sex ratio (anxiety, 18 males; nonanxiety, 23 males; *P* = .92) or age (anxiety, mean age ± SD = 47.14 ± 20.17 years; nonanxiety, mean age ± SD = 53.93 ± 8.31 years; *P* = .16). Lesions in the anxiety group showed significant functional connectivity within the anxiety network (Figure 3, *t* = 2.58; *P* = .02), while lesions in the nonanxiety group did not (Figure 3, *t* = 1.30; *P* = .21). The average connectivity strength was also significantly higher for anxiety-associated lesions than nonanxiety-associated lesions (Figure 3, anxiety, mean ± SD = 6.37 ± 11.58; nonanxiety, mean ± SD = −2.02 ± 8.24; *t* = 2.99; *P* = .004).

### 3.4. Relevance to rTMS Sites Alleviating Anxiety Symptom

**Figure 4:**
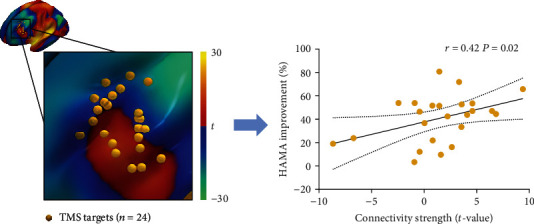
Repetitive transcranial magnetic stimulation (rTMS) targeting the anxiety network improves clinical anxiety symptoms. The connectivity strength of rTMS targets within the anxiety network (*n* = 24) was positively correlated with HAMA score improvement after treatment (*r* = .42, *P* = .02). rTMS = repetitive transcranial magnetic stimulation, HAMA = Hamilton Anxiety Scale.

To assess the clinical significance of this anxiety network, we retrospectively analyzed the clinical findings of 24 patients with anxiety symptoms receiving rTMS treatment (HAMA ≥ 14; 14 females; 23.42 ± 7.51 years of age; 13.71 ± 1.9 years of education; 3.46 ± 4.19 years illness duration). The connectivity strength of rTMS targets extracted from the anxiety network map was positively correlated with anxiety symptom improvement (Figure 4, *r* = .42; *P* = .02), and this correlation remained after correcting for covariates (sex, age, illness duration, and education; *r* = .43, *P* = .03) and when using a smaller seed size (3 mm seed radius instead of 6 mm; *r* = .41, *P* = .02). The correlation with functional connectivity strength (*t*-value) was still significant when using two other measures, see details in Supplementary S5 and Supplementary Figure 5.

## 4. Discussion

Using coordinate network mapping, we explored the pathophysiology of anxiety disorders at the network level and demonstrated the clinical significance of our findings. First, we found that spatially heterogenous sites of brain atrophy in anxiety disorder patients are mapped to a common functional network defined by a hub region in the amygdala. Second, this common network overlapped strongly with lesions causing anxiety symptoms. Finally, the connectivity strength of rTMS targets within the anxiety network predicted symptom improvement, suggesting potential therapeutic relevance.

Neurological and psychiatric symptoms stem from dysfunction in single brain regions as well as disruption of cooperative activity among functionally associated areas [23]. This holds true for anxiety as well, as emergence is linked to the coordinated activity of multiple regions across the brain [30, 31]. Traditional meta-analysis can identify local brain regions most consistently abnormal or otherwise associated with anxiety but neglect the contribution of connectivity disruption among brain regions. In this study, we attempted to reconcile the heterogenous VBM findings of past studies with shared symptoms using CNM. In accordance with studies applying CNM to other psychiatric and neurological disorders [20–22, 32, 33], we identified a common anxiety network associated with spatially heterogenous abnormalities revealed by VBM [34–36]. This anxiety network was defined by hub regions in the bilateral amygdala and included positive connectivity among regions consistently implicated in psychiatry disorders, such as the ventrolateral prefrontal cortex, insula, and cingulate cortex [22, 37].

The amygdala is involved in emotion recognition, learning, and processing, and also regulates cognitive processes such as the encoding of memories with emotional salience [38, 39]. Our findings emphasize the role of amygdala connectivity in anxiety, consistent with meta-analyses reporting abnormal local structure [3], function [31], and activity in the amygdala during anxiety expression [30]. The hub region of the identified anxiety network was localized primarily in the SFA (containing the anterior, ventral, and posterior sections), a structure crucial for adaptively responding to environmental threats [40, 41]. Thus, the SFA may be a critical component of the neural network underlying anxiety and anxiety disorders. For instance, reduced SFA connectivity with perceptual systems may heighten sensitivity to environmental stimuli and thereby increase susceptibility to anxiety [42], while normalizing SFA connectivity may serve to alleviate anxiety [43].

Identifying brain lesions associated with specific psychiatric or neurological symptoms is a powerful approach for localizing human brain functions and promising treatment targets [24, 44]. To investigate the clinical significance of our anxiety network, we mapped the locations of brain lesions associated with emergent anxiety and found significantly greater spatial alignment compared to lesions not associated with anxiety. This finding provides strong evidence for a causal link between our network and anxiety symptoms [24]. Confirming this causal relationship has important implications for optimizing clinical treatments, such as rTMS.

Transcranial magnetic stimulation is a neuromodulation technique with demonstrated efficacy for various psychiatric disorders, but with notably limited effects on anxiety [10]. Many clinical trials on TMS interventions for anxiety have targeted the bilateral dorsolateral prefrontal cortex [11] but target location has varied considerably [45–47] as there is no consensus on the underlying neural correlates. In this study, we identified a common anxiety network that can explain the heterogenous findings of previous structural imaging studies. More importantly, retrospective clinical outcome aligns with this network. This indicates that the network may provide beneficial guidance for rTMS treatment. Translation of this population-level network to the individual patient level may further enhance targeting accuracy and thus the clinical efficacy of rTMS.

This study has several limitations. First, as in previous CNM studies, we created spheres centered on each atrophy coordinate from individual VBM studies and combined them to create a functional connectivity map. However, the real atrophy map may have continuously extended broadly across brain regions, so a portion of atrophy signals could have been missed in the sphere seeds. Second, the number of studies contributing to the anxiety network (*n* = 16) was relatively small, so the network defined could be common to multiple disorders characterized by anxiety (e.g., generalized anxiety disorder, panic disorder, social anxiety disorder, and specific phobia). As the number of VBM studies increases, future studies may define more specific networks for each diagnose. Third, we identified brain regions with overlap rates of 80% rather than the 90% or even 100% in previous lesion network mapping studies [23]. This lower overlap is expected given that functional neuroimaging studies are more vulnerable to methodological differences and imaging noise than lesion studies. However, this reduced overlap should bias against finding a common brain network.

## 5. Conclusions

Using a coordinate network mapping approach, we show that regions of decreased gray matter volume in anxiety disorders localize to a common anxiety network defined by connectivity to a hub primarily located in the superficial amygdala. The causal relationship of the anxiety network with clinical anxiety symptom was validated by independent lesion data. More importantly, we found that the functional connectivity between rTMS target and anxiety network can predict therapeutic efficacy, suggesting that this anxiety network can be used to identify optimal rTMS targets for anxiety disorders.

## Figures and Tables

**Table 1:** Participant characteristics.

Characteristic	Healthy cohort (*n* = 652)	Anxiety cohort (*n* = 108)	Lesion cohort		rTMS cohort	
			Anxiety (*n* = 22)	Nonanxiety (*n* = 28)	Pre (*n* = 24)	Post (*n* = 24)
Sex (M/F)	316/336	40/68	18/4	23/5	10/14	
Mean age (years)	22.9 ± 5.55	35.33 ± 12.37	47.14 ± 20.17	53.93 ± 8.31	23.42 ± 7.51	
Mean HAMA	─	19.31 ± 5.17	25.6 ± 4.28^∗^	3.11 ± 3.9	28.71 ± 5.56	16.88 ± 6.58

Notes: results expressed as mean ± standard deviation. rTMS = repetitive transcranial magnetic stimulation, HAMA = Hamilton Anxiety Scale. ⁣^∗^Data based on five patients.

## Data Availability

The data that support the findings of this study are available from the corresponding author upon reasonable request.

## Supplementary Materials

Supplementary Materials Supplementary Methods: Supplementary S1: the inclusion criteria for articles reporting gray matter volume reduction (atrophy) in anxiety disorder patients. Supplementary S2: the details of the healthy connectome, including detailed scan sequences used for the healthy connectome, along with data processing information. Supplementary S3: the inclusion and exclusion criteria used in studies involving nonanxiety disorders. Supplementary S4: the sources of 50 patients with brain lesions. Supplementary S5: the detailed rTMS protocol for the 24 patients exhibiting anxiety symptoms who underwent a 14-day rTMS treatment. Supplementary Tables: Table S1: the search strategies used in studies reporting gray matter volume reduction in patients with anxiety disorders. Table S2: the search strategies employed in studies reporting brain lesion patients with anxiety symptoms. Table S3: studies reporting gray matter volume reduction in anxiety patients. Table S4: the meta-analyses used to identify nonanxiety disorders studies. Table S5: the studies reporting gray matter volume reduction in nonanxiety patients. Supplementary Figures: Supplementary Figure 1: the work flow used to identify anxiety patients with gray matter atrophy from the literature. Supplementary Figure 2: the results of the robust test of sensitivity map. Supplementary Figure 3: the results of a robust test for localizing the anxiety network using a sensitivity map overlap threshold of 75% (12 out of 16). Supplementary Figure 4: the work flow used to identify anxiety-causing lesions from the literature. Supplementary Figure 5: correlation analysis between connectivity strength of rTMS targets and two other measures: (1) pretreatment HAMA score − posttreatment HAMA score and (2) posttreatment HAMA score (with pretreatment HAMA score as a covariate).
