# Supplementary material for: Heterogeneous Brain Atrophy Sites in Anxiety Disorders Map to a Common Brain Network
Source: Depress Anxiety. 2024 Apr 2;2024:3827870. doi: 10.1155/2024/3827870 (PMC11919243; doi:10.1155/2024/3827870)
Supplement: Supplementary Materials — Supplementary Methods: Supplementary S1: the inclusion criteria for articles reporting gray matter volume reduction (atrophy) in anxiety disorder patients. Supplementary S2: the details of the healthy connectome, including detailed scan sequences used for the healthy connectome, along with data processing information. Supplementary S3: the inclusion and exclusion criteria used in studies involving nonanxiety disorders. Supplementary S4: the sources of 50 patients with brain lesions. Supplementary S5: the detailed rTMS protocol for the 24 patients exhibiting anxiety symptoms who underwent a 14-day rTMS treatment. Supplementary Tables: Table S1: the search strategies used in studies reporting gray matter volume reduction in patients with anxiety disorders. Table S2: the search strategies employed in studies reporting brain lesion patients with anxiety symptoms. Table S3: studies reporting gray matter volume reduction in anxiety patients. Table S4: the meta-analyses used to identify nonanxiety disorders studies. Table S5: the studies reporting gray matter volume reduction in nonanxiety patients. Supplementary Figures: Supplementary Figure 1: the work flow used to identify anxiety patients with gray matter atrophy from the literature. Supplementary Figure 2: the results of the robust test of sensitivity map. Supplementary Figure 3: the results of a robust test for localizing the anxiety network using a sensitivity map overlap threshold of 75% (12 out of 16). Supplementary Figure 4: the work flow used to identify anxiety-causing lesions from the literature. Supplementary Figure 5: correlation analysis between connectivity strength of rTMS targets and two other measures: (1) pretreatment HAMA score − posttreatment HAMA score and (2) posttreatment HAMA score (with pretreatment HAMA score as a covariate). [file 3827870.f1.docx]

**SUPPLEMENTARY MATERIALS**

**Heterogeneous brain atrophy sites in anxiety disorders map to a common brain network**

|  |  |
| --- | --- |

[Supplementary Methods 2](#_Toc161066600)

[Supplementary S1: Inclusion criteria 2](#_Toc161066601)

[Supplementary S2: Details of the healthy connectome 2](#_Toc161066602)

[Supplementary S3: Specificity testing 3](#_Toc161066603)

[Supplementary S4: Anxiety related lesions 3](#_Toc161066604)

[Supplementary S5: Relevance of the anxiety network for treatment response 4](#_Toc161066605)

[Supplementary Tables 5](#_Toc161066606)

[Table S1: Search strategies for studies reporting gray matter volume reduction in anxiety patients 5](#_Toc161066607)

[Table S2: Search strategies for studies reporting brain lesion patients with anxiety symptoms 6](#_Toc161066608)

[Table S3: Studies reporting gray matter volume reduction in anxiety patients 7](#_Toc161066609)

[Table S4: Meta-analyses used to identify non-anxiety disorders studies 8](#_Toc161066610)

[Table S5: Studies reporting gray matter volume reduction in non-anxiety patients 9](#_Toc161066611)

[Supplementary Figures 12](#_Toc161066612)

[Supplementary Figure 1: Work flow for identifying anxiety patients with gray matter atrophy from the literature. 12](#_Toc161066613)

[Supplementary Figure 2: The robust test of sensitivity map 13](#_Toc161066614)

[Supplementary Figure 3: The robust test of anxiety network localization at a sensitivity map overlap threshold of 75% (12 of 16). 14](#_Toc161066615)

[Supplementary Figure 4: Work flow for identifying anxiety-causing lesions from the literature. 15](#_Toc161066616)

[Supplementary Figure 5: Another two measures of clinical outcomes (besides the improvement rate) showing significant correlations with the functional connectivity strength of target (t-value). 16](#_Toc161066617)

[Reference 17](#_Toc161066618)

**Supplementary Methods**

**Supplementary S1: Inclusion criteria**

The included studies reporting gray matter volume reduction in anxiety patients had to meet the following criteria: 1) articles were limited to English and human, 2) studies used whole-brain voxel-based morphometry (VBM) analysis, 3) patients fulfilling DSM criteria for at least one of the anxiety disorders (e.g., generalized anxiety disorder, panic disorder, social anxiety disorder and specific phobia), 4) studies contained a comparison between patients with anxiety disorders and healthy controls, 5) peak coordinates reported in Montreal Neurological Institute (MNI) or Talairach space.

Studies were excluded if: 1) they were theoretical papers, reviews, meta-analyses and case reports, 2) patients had current comorbid neurological or psychiatric disorders other than anxiety disorders, 3) results were not significant after whole-brain correction.

**Supplementary S2: Details of the healthy connectome**

A total of 652 healthy adults were included in this study, all participants provided written informed consent before the experiments. High-resolution structural images of brain anatomy and resting-state functional MRI (rs-fMRI) data were acquired for all individuals. The exclusion criteria were: 1) history of significant head trauma, 2) alcohol or drug abuse, 3) focal brain lesions on T1- or T2-weighted fluid-attenuated inversion-recovery magnetic resonance images, 4) head motion exceeding 3 mm in translation or 3° in rotation during rs-fMRI scanning, or 5) Hamilton Anxiety Rating Scale (HAMA) or Hamilton Depression Rating Scale (HAMD) scores > 7.

Structural and fMRI data were obtained at the XXXXXXX with a 3-T scanner (Discovery 750; GE Healthcare, Milwaukee, WI, USA). High-resolution T1-weighted images were acquired in the sagittal orientation using a magnetization prepared rapid gradient-echo sequence (repetition/echo time, 8.16/3.18 ms; flip angle, 12; field of view, 256 × 256 mm^2^; 256 × 256 matrices; section thickness, 1 mm; voxel size, 1 × 1 × 1 mm^3^). Resting-state functional images were acquired using a single shot gradient-recalled echo planar imaging sequence (repetition/echo time, 2400/30 ms; flip angle, 90; field of view, 192 × 192 mm^2^; 64 × 64 in-plane matrix; section thickness, 3 mm; voxel size, 3 × 3 × 3 mm^3^; 46 transverse sections). A total of 217 volumes were acquired (~8.7 min). During scanning, participants were instructed to rest with their eyes closed without falling asleep.

The rs-fMRI data were preprocessed using SPM12 software (<https://www.fil.ion.ucl.ac.uk/spm/>) and ANFI (<https://afni.nimh.nih.gov/afni/>). The processing steps were as follows: 1) delete the first 5 time points, 2) remove temporal spikes more than 2.5 SDs from the mean (AFNI’s 3dDespike), 3) slice timing correction, 4) head motion correction, 5) co-registration to the structural image, 6) regress out nuisance regressors (24 head motion parameters, and average signals in the cerebrospinal fluid, white matter, and whole brain), 7) spatial normalization to the MNI space using the matrix produced by structural image segmentation using DARTEL algorithm in SPM12 [1], and 8) spatial smoothing with a 4 mm full width at half maximum Gaussian kernel.

**Supplementary S3: Specificity testing**

The random *t* maps were generated as the study-specific *t* maps, but randomly re-distributed the coordinates within the gray matter. The non-anxiety disorders included six additional mental disorders for which there was no significant genetic association with anxiety [2], including autism spectrum disorder, Tourette's syndrome, anorexia nervosa, bipolar disorder, obsessive-compulsive disorder and post-traumatic stress disorder. We utilized existing meta-analyses to identify non-anxiety disorders studies.

The included studies had to meet the following criteria: 1) articles were limited to English and human, 2) studies used whole-brain VBM analysis, 3) patients fulfilling DSM criteria for anorexia nervosa (AN), autism spectrum disorder (ASD), bipolar disorder (BD), obsessive-compulsive disorder (OCD), post-traumatic stress disorder (PTSD) and Tourette syndrome (TS), 4) studies contained a comparison between patients and healthy controls (HC), 5) compared with HC, patients showed decreased gray matter volume or gray matter density, 6) peak coordinates reported in MNI or Talairach space. Studies were excluded if: 1) they were theoretical papers, reviews, meta-analyses and case reports, 2) results were not significant after whole-brain correction. Meta-analyses and individual studies are available in Supplementary Table S4 and S5, respectively.

The comparisons between connectivity maps of anxiety coordinates with random maps as well as with maps of non-anxiety disorders were performed by voxel-wise two-sample *t*-tests (cluster-forming threshold at voxel-level *P* < .001, cluster-level FWE-corrected *P_FWE_* < .05) using the SnPM tool in SPM12.

**Supplementary S4: Anxiety related lesions**

A total of 50 patients with brain lesion were included in our study. We retrospectively used clinical data from 33 patients with brain lesion, 5 of whom developed anxiety symptoms after lesion (HAMA ≥ 14) and 28 of whom did not. Due to the shortage of lesion patients with anxiety symptoms, we conducted searches on the PubMed and Web of Science databases for patients who developed anxiety symptoms following brain lesion. The search strategy and flow chart are available in Supplementary Figure S4 and Table S2. We identified 17 patients who met the inclusion criteria [3, 4, 5, 6, 7, 8, 9, 10, 11, 12, 13, 14, 15, 16, 17, 18, 19].

**Supplementary S5: Relevance of the anxiety network for treatment response**

Twenty-four patients with anxiety symptoms underwent a 14-day rTMS intervention, their detailed rTMS protocol is shown below.

A MagStim Rapid^2^ stimulator (MagStim Company Ltd.) was used with a 70-mm air-cooled figure-of-eight coil for intermittent theta-burst stimulation (iTBS) treatment. Treatment was based on a protocol by Huang and colleagues [20]: the main mode of iTBS is to stimulate 2 seconds, at 8-second intervals for 20 cycles. One session of iTBS was 190 seconds in duration, which consisted of 3-pulse bursts at 50 Hz repeated every 200 milliseconds at 5 Hz until a total of 600 pulses was reached [21]. The researchers delivered iTBS at 80% of the resting motor threshold (RMT) or the highest intensity the stimulator could deliver for this protocol (50% of maximum output) [22]. During treatment, all patients sat in chairs, and the TMS coil was fixed to the head with a MagStim Articulated Coil Stand (MagStim Company Ltd.). We adjusted the handle orientation of the coil backward or outward to ensure that it was perpendicular to the gyrus where the target was located. Participants wore sound-attenuating earplugs to prevent hearing damage. To achieve cumulative aftereffects, this protocol was repeated 3 times (1800 pulses in total), with each occurrence separated by two 15-minute breaks (controlled by a stopwatch), which is in line with previous methodological studies. During the 15-minute treatment interval, all patients were silent and rested with their eyes closed [23]. The target for each patient was determined based on the records in the neuronavigation software.

In response to the reviewers' questions, we also calculated the correlation with functional connectivity strength (*t*-value) using two other measures: 1) pre-treatment HAMA score - post-treatment HAMA score and 2) post-treatment score (with pre-treatment HAMA score as a covariate) (the results are available in Supplementary Figure S5).

**Supplementary Tables**

**Table S1: Search strategies for studies reporting gray matter volume reduction in anxiety patients**

| Database | PubMed |
| --- | --- |
| Date of search | 2022.10.31 |
| Search strategy | **Search term:**  (anxiety[Title/Abstract] OR nervousness[Title/Abstract] OR anxious[Title/Abstract]) AND ("voxel-based morphometry"[Title/Abstract] OR VBM[Title/Abstract] OR "gray matter"[Title/Abstract] OR "gray matter atrophy"[Title/Abstract] OR "gray matter volume"[Title/Abstract] OR "gray matter concentration"[Title/Abstract])  **Publication data:** up to 2022.10.31  **Filters:** Humans, English |
| Items found | 475 |

| Database | Web of Science |
| --- | --- |
| Date of search | 2022.10.31 |
| Search strategy | **Search term:**  (anxiety OR nervousness OR anxious) (Topic) AND ("voxel-based morphometry" OR VBM OR "gray matter" OR "gray matter atrophy" OR "gray matter volume" OR "gray matter concentration") (Topic) and English (Language)  **Publication data:** up to 2022.11.8 |
| Items found | 1059 |

| Database | PubMed |
| --- | --- |
| Date of search | 2022.10.20 |
| Search strategy | **Search term:**  (CT OR "computerized tomography" OR "computed tomography" OR MRI OR "Magnetic resonance" OR "magnetic resonance imaging" OR neuroimaging) AND (anxiety OR nervousness OR anxious OR hypervigilance OR phobia)AND (brain lesion[Title/Abstract] OR brain damage[Title/Abstract] OR white matter abnormalities[Title/Abstract] OR gray matter abnormalities[Title/Abstract] OR brain abnormalities[Title/Abstract] OR spinal injur*[Title/Abstract] OR spinal lesion*[Title/Abstract] OR brain injur*[Title/Abstract] OR head injur*[Title/Abstract] OR craniocerebral trauma[Title/Abstract] OR central nervous system infection[Title/Abstract] OR central nervous system neoplasms[Title/Abstract] OR brain metastasis[Title/Abstract] OR neurosurgical procedure[Title/Abstract] OR multiple sclerosis[Title/Abstract] OR encephalitis[Title/Abstract] OR cerebrovascular disorder[Title/Abstract] OR intracranial hemorrhage[Title/Abstract] OR ischem*[Title/Abstract] OR apoplex*[Title/Abstract] OR insult*[Title/Abstract] OR stroke[Title/Abstract] OR nervous system malformation[Title/Abstract])  **Publication data:** up to 2022.10.20  **Filters:** Humans, English |
| Items found | 604 |

**Table S2: Search strategies for studies reporting brain lesion patients with anxiety symptoms**

| Database | Web of science |
| --- | --- |
| Date of search | 2022.10.31 |
| Search strategy | **Search term:**  (CT OR "computerized tomography" OR "computed tomography" OR MRI OR "Magnetic resonance" OR "magnetic resonance imaging" OR neuroimaging) ( Topic ) AND (anxiety OR nervousness OR anxious OR hypervigilance OR phobia) ( Topic ) AND (brain lesion OR brain damage OR white matter abnormalities OR gray matter abnormalities OR brain abnormalities OR spinal injur* OR spinal lesion* OR brain injur* OR head injur* OR craniocerebral trauma OR central nervous system infection OR central nervous system neoplasms OR brain metastasis OR neurosurgical procedure OR multiple sclerosis OR encephalitis OR cerebrovascular disorder OR intracranial hemorrhage OR ischem* OR apoplex* OR insult* OR stroke OR nervous system malformation) (Topic)  **Publication data:** up to 2022.10.31  **Filters:** English |
| Items found | 1532 |

**Table S3: Studies reporting gray matter volume reduction in anxiety patients**

| **Reference** | **Diagnose** | **Anxiety** | | | **Healthy controls** | | **P_(corr)_** |
| --- | --- | --- | --- | --- | --- | --- | --- |
|  |  | **Sample(M/F)** | **Age** | **Duration**  **(year)** | **Sample(M/F)** | **Age** |  |
| **Makovac[24]** | **GAD** | **19(3/16)** | **30** | **16.78** | **19(3/16)** | **29.2** | **<0.05** |
| **Ma[25]** | **GAD** | **21(12/9)** | **34.92** | **2.39** | **20(10/10)** | **35.96** | **<0.001** |
| **Chen[26]** | **GAD** | **72(31/41)** | **39.04** | **4.56** | **57(27/30)** | **40.91** | **<0.05** |
| **Massana[27]** | **PD** | **18(7/11)** | **36.8** | **NA** | **18(8/10)** | **36.7** | **<0.05** |
| **Yoo[28]** | **PD** | **18(9/9)** | **33.3** | **3.6** | **18(11/7)** | **32** | **<0.05** |
| **Hayano[29]** | **PD** | **27(10/17)** | **38.2** | **5.4** | **30(9/21)** | **35.3** | **<0.05** |
| **Lai[30]** | **PD** | **30(11/19)** | **47.03** | **NA** | **21(10/11)** | **41.14** | **<0.05** |
| **Na[31]** | **PD** | **12(5/7)** | **43.08** | **NA** | **22(11/11)** | **40.18** | **<0.05** |
| **Lai[32]** | **PD** | **53(25/28)** | **43.28** | **0.45** | **54(25/29)** | **40.38** | **<0.05** |
| **Wu[33]** | **PD** | **24(12/12)** | **31.6** | **2.9** | **22(11/11)** | **33.1** | **<0.05** |
| **Liao[34]** | **SAD** | **18(12/6)** | **22.67** | **4.1** | **18(13/5)** | **21.89** | **<0.05** |
| **Talati[35]** | **SAD** | **17(6/11)** | **29.1** | **NA** | **17(7/10）** | **31.3** | **<0.05** |
| **Meng[36]** | **SAD** | **20(14/6)** | **21.8** | **4.21** | **19(13/6)** | **21.58** | **<0.05** |
| **Zhao[37]** | **SAD** | **24(15/9)** | **24.5** | **7.6** | **41(26/15)** | **27.1** | **<0.001** |
| **Zhang[38]** | **SAD** | **49(30/19)** | **24.6** | **7.2** | **53(31/22)** | **23.4** | **<0.05** |
| **Rivero[39]** | **SP** | **31(6/25)** | **35.16** | **NA** | **31(7/24)** | **22** | **<0.05** |

Note-both age and disease duration are presented as means. The corrected *P* value refers to the significance of the difference in gray matter between the two groups. GAD = generalized anxiety disorder, PD = panic disorder, SAD = social anxiety disorder, SP = specific phobia

**Table S4: Meta-analyses used to identify non-anxiety disorders studies**

| **Year** | **Author** | **Diagnose** |
| --- | --- | --- |
| **2022** | **Sader[40]** | **Anorexia nervosa (AN)** |
| **2020** | **Lukito[41]** | **Autism spectrum disorder (ASD)** |
| **2021** | **Gong[42]** | **Bipolar disorder (BD)** |
| **2020** | **Pico[43]** | **Obsessive-compulsive disorder (OCD)** |
| **2021** | **Serra[44]** | **Post-traumatic stress disorder (PTSD)** |
| **2021** | **Wan[45]** | **Tourette syndrome (TS)** |

Note-the studies included from these meta-analyses are shown in Table S5.

**Table S5: Studies reporting gray matter volume reduction in non-anxiety patients**

| **Reference** | **Diagnose** | **Patients** | | **Healthy controls** | | **P_(corr)_** |
| --- | --- | --- | --- | --- | --- | --- |
|  |  | **Sample(M/F)** | **Age** | **Sample(M/F)** | **Age** |  |
| **Castro[46]** | **AN** | **12(1/11)** | **14.5** | **9(1/8)** | **14.6** | **<0.05** |
| **Suchan[47]** | **AN** | **15(0/15)** | **26.8** | **15(0/15)** | **29.5** | **<0.05** |
| **Boghi[48]** | **AN** | **21(0/21)** | **29** | **27(0/27)** | **30.8** | **<0.05** |
| **Brooks[49]** | **AN** | **14(0/14)** | **26** | **21(0/21)** | **26** | **<0.05** |
| **Gaudio[50]** | **AN** | **16(0/16)** | **15.2** | **16(0/16)** | **15.1** | **<0.05** |
| **Joos[51]** | **AN** | **12(0/12)** | **25** | **18(0/18)** | **26.9** | **<0.05** |
| **Friederich[52]** | **AN** | **12(0/12)** | **24.3** | **14(0/14)** | **25.6** | **<0.05** |
| **Fonville[53]** | **AN** | **31(0/31)** | **23** | **31(0/31)** | **25** | **<0.05** |
| **Bar[54]** | **AN** | **26(3/23)** | **22.96** | **26(3/23)** | **24** | **<0.05** |
| **DAgata[55]** | **AN** | **21(0/21)** | **21** | **17(0/17)** | **23** | **<0.05** |
| **Fujisawa[56]** | **AN** | **20(0/20)** | **14.15** | **14(0/14)** | **14.93** | **<0.05** |
| **Seitz[57]** | **AN** | **56(0/56)** | **15.47** | **50(0/50)** | **15.75** | **<0.01** |
| **Vanopstal[58]** | **AN** | **10(0/10)** | **22.1** | **11(0/11)** | **20.8** | **<0.05** |
| **Kohmura[59]** | **AN** | **23(0/23)** | **28.5** | **29(0/29)** | **28.2** | **<0.05** |
| **Martin[60]** | **AN** | **26(0/26)** | **16.5** | **20(0/20)** | **17.25** | **<0.05** |
| **Nickel[61]** | **AN** | **34(0/34)** | **23.8** | **41(0/41)** | **23.6** | **<0.001** |
| **Boddaert[62]** | **ASD** | **21(16/5)** | **9.3** | **12(7/5)** | **10.8** | **<0.05** |
| **McAlonan[63]** | **ASD** | **33(27/6)** | **11.6** | **55(47/8)** | **10.7** | **<0.002** |
| **Kosaka[64]** | **ASD** | **32(32/0)** | **23.8** | **40(40/0)** | **22.5** | **<0.05** |
| **Cheng[65]** | **ASD** | **25(25/0)** | **13.7** | **25(25/0)** | **13.5** | **<0.05** |
| **Hyde[66]** | **ASD** | **15(15/0)** | **22.7** | **15(15/0)** | **19.2** | **<0.05** |
| **Kurth[67]** | **ASD** | **52(38/14)** | **11.2** | **52(38/14)** | **11.14** | **<0.05** |
| **Riva[68]** | **ASD** | **26(23/3)** | **5.83** | **21(13/8)** | **6.83** | **<0.05** |
| **Sato[69]** | **ASD** | **36(25/11)** | **27** | **36(25/11)** | **24.9** | **<0.05** |
| **Ni[70]** | **ASD** | **28(28/0)** | **12** | **61(61/0)** | **12.4** | **<0.01** |
| **Yang[71]** | **ASD** | **16(10/6)** | **10.44** | **16(10/6)** | **10.5** | **<0.05** |
| **Lochhead[72]** | **BD** | **11(6/5)** | **38.2** | **31(16/15)** | **36** | **<0.05** |
| **Lyoo[73]** | **BD** | **39(16/23)** | **38.3** | **43(19/24)** | **35.7** | **<0.05** |
| **Nugent[74]** | **BD** | **36(10/26)** | **39.2** | **65(19/46)** | **38** | **<0.05** |
| **Chen[75]** | **BD** | **24(6/18)** | **38.21** | **25(7/18)** | **38.44** | **<0.05** |
| **Haldane[76]** | **BD** | **44(20/24)** | **42.7** | **44(20/24)** | **43.1** | **<0.01** |
| **Almeida[77]** | **BD** | **27(10/17)** | **31.89** | **28(13/15)** | **30.82** | **<0.05** |
| **Ha[78]** | **BD** | **23(8/15)** | **35.2** | **23(8/15)** | **36** | **<0.05** |
| **Ha[78]** | **BD** | **23(8/15)** | **35.6** | **23(8/15)** | **36** | **<0.05** |
| **Stanfield[79]** | **BD** | **66(30/36)** | **36.4** | **66(31/35)** | **39** | **<0.05** |
| **Tost[80]** | **BD** | **42(19/23)** | **42.4** | **42(19/23)** | **42.2** | **<0.05** |
| **Brown[81]** | **BD** | **15(7/8)** | **46.2** | **21(10/11)** | **45** | **<0.05** |
| **Frangou[82]** | **BD** | **47(21/26)** | **46.2** | **71(36/35)** | **39.8** | **<0.05** |
| **Hajek[83]** | **BD** | **12(6/6)** | **45.6** | **11(3/8)** | **46** | **<0.05** |
| **Ambrosi[84]** | **BD** | **20(5/15)** | **41.95** | **21(6/15)** | **34.61** | **<0.05** |
| **Redlich[85]** | **BD** | **58(21/37)** | **37.5** | **58(21/37)** | **37.7** | **<0.05** |
| **Sarıçiçek[86]** | **BD** | **28(8/20)** | **36.3** | **29(13/16)** | **33.6** | **<0.05** |
| **Shepherd[87]** | **BD** | **30(12/18)** | **39.06** | **34(16/18)** | **32.6** | **<0.05** |
| **Alonso[88]** | **BD** | **33(18/15)** | **44.13** | **28(12/16)** | **44.01** | **<0.05** |
| **Matsubara[89]** | **BD** | **10(3/7)** | **46.9** | **27(10/17)** | **48.3** | **<0.05** |
| **Poletti[90]** | **BD** | **206(72/134)** | **46.15** | **136(68/68)** | **33.31** | **<0.05** |
| **Sani[91]** | **BD** | **78(38/40)** | **44.56** | **78(38/40)** | **44.38** | **<0.05** |
| **Altamura[92]** | **BD** | **46(23/23)** | **33.67** | **56(29/27)** | **25.3** | **<0.001** |
| **Lee[93]** | **BD** | **21(7/14)** | **37** | **21(7/14)** | **37** | **<0.05** |
| **Maggioni[94]** | **BD** | **176(69/107)** | **44.7** | **383(195/188)** | **30.4** | **<0.05** |
| **Wang[95]** | **BD** | **30(15/15)** | **36.3** | **31(18/13)** | **33.61** | **<0.05** |
| **Lee[96]** | **BD** | **65(29/36)** | **35.06** | **65(28/37)** | **34.52** | **<0.017** |
| **Li[97]** | **BD** | **44(18/26)** | **23.11** | **36(22/14)** | **22.78** | **<0.05** |
| **Song[98]** | **BD** | **36(16/20)** | **30.6** | **29(10/19)** | **29.3** | **<0.01** |
| **Vai[99]** | **BD** | **74(19/55)** | **47.26** | **74(35/39)** | **36.38** | **<0.05** |
| **Pujol[100]** | **OCD** | **72(40/32)** | **29.8** | **72(40/32)** | **30.1** | **<0.05** |
| **Valente[101]** | **OCD** | **19(10/9)** | **32.7** | **15(7/8)** | **32.3** | **<0.05** |
| **Gilbert[102]** | **OCD** | **25(13/12)** | **37.5** | **20(9/11)** | **29.8** | **<0.05** |
| **Koprivova[103]** | **OCD** | **14(5/9)** | **28.6** | **15(6/9)** | **28.7** | **<0.05** |
| **Togao[104]** | **OCD** | **23(9/14)** | **32.6** | **26(12/14)** | **31.3** | **<0.05** |
| **Hou[105]** | **OCD** | **33(18/15)** | **25.3** | **33(18/15)** | **25** | **<0.05** |
| **Subira[106]** | **OCD** | **30(20/10)** | **32.23** | **95(55/40)** | **33.92** | **<0.05** |
| **Hashimoto[107]** | **OCD** | **24(11/13)** | **35.7** | **30(14/16)** | **32.5** | **<0.05** |
| **Hashimoto[107]** | **OCD** | **15(7/8)** | **32.5** | **30(14/16)** | **32.5** | **<0.05** |
| **Tang[108]** | **OCD** | **26(15/11)** | **25.5** | **32(17/15)** | **26.2** | **<0.05** |
| **Tang[109]** | **OCD** | **18(11/7)** | **27.3** | **16(10/6)** | **26.8** | **<0.05** |
| **Moreira[110]** | **OCD** | **40(13/27)** | **26.28** | **40(13/27)** | **26.45** | **<0.05** |
| **Moon[111]** | **OCD** | **18(11/7)** | **27.6** | **18(11/7)** | **30.7** | **<0.05** |
| **Corbo[112]** | **PTSD** | **14(6/8)** | **33.36** | **14(6/8)** | **33.29** | **<0.05** |
| **Chen[113]** | **PTSD** | **12(4/8)** | **34.56** | **12(4/8)** | **33.25** | **<0.05** |
| **Li[114]** | **PTSD** | **12(4/8)** | **34.56** | **12(4/8)** | **33.25** | **<0.001** |
| **Bryant[115]** | **PTSD** | **7(NA)** | **NA** | **13(NA)** | **NA** | **<0.05** |
| **Bryant[115]** | **PTSD** | **6(NA)** | **NA** | **13(NA)** | **NA** | **<0.05** |
| **Felming[116]** | **PTSD** | **21(NA)** | **NA** | **17(NA)** | **NA** | **<0.05** |
| **Sui[117]** | **PTSD** | **13(0/13)** | **24.46** | **13(0/13)** | **26** | **<0.001** |
| **Sui[118]** | **PTSD** | **11(0/11)** | **25.55** | **8(0/8)** | **27.5** | **<0.005** |
| **Zhang[119]** | **PTSD** | **10(10/0)** | **40.8** | **10(10/0)** | **34.3** | **<0.05** |
| **Nardo[120]** | **PTSD** | **15(12/3)** | **43.33** | **17(11/6)** | **41.59** | **<0.05** |
| **Rocha[121]** | **PTSD** | **16(7/9)** | **43.3** | **16(7/9)** | **44.9** | **<0.05** |
| **Tavanti[122]** | **PTSD** | **25(8/17)** | **38.16** | **25(8/17)** | **38.08** | **<0.05** |
| **Tan[123]** | **PTSD** | **12(12/0)** | **37.6** | **14(14/0)** | **40.9** | **<0.05** |
| **Cheng[124]** | **PTSD** | **30(21/9)** | **26.3** | **30(21/9)** | **26.2** | **<0.05** |
| **Bossini[125]** | **PTSD** | **19(10/9)** | **40** | **19(15/4)** | **41** | **<0.05** |
| **ODoherty[126]** | **PTSD** | **25(12/13)** | **34** | **25(12/13)** | **31.7** | **<0.01** |
| **Wittfoth[127]** | **TS** | **29(29/0)** | **30.7** | **24(24/0)** | **30.6** | **<0.05** |

Note-both age and disease duration are presented as means. The corrected *P* value refers to the significance of the difference in gray matter between the two groups. AN = Anorexia nervosa, ASD = Autism spectrum disorder, BD = Bipolar disorder, OCD = Obsessive-compulsive disorder, PTSD = Post-traumatic stress disorder, TS = Tourette syndrome

**Supplementary Figures**

**Supplementary Figure 1: Work flow for identifying anxiety patients with gray matter atrophy from the literature.**


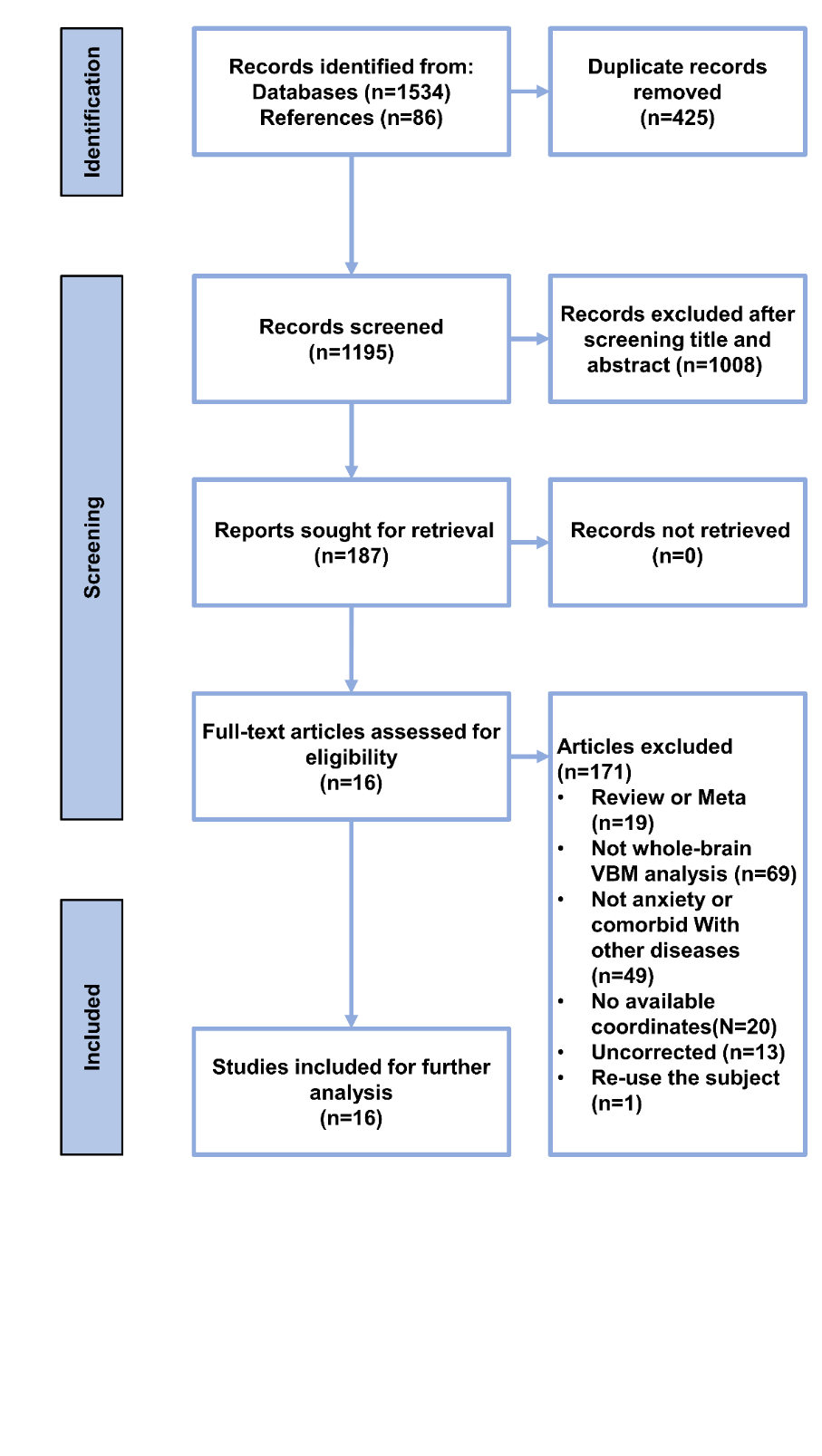


**Supplementary Figure 1:** 1620 articles matched initial search criteria. After eliminating duplicate articles, the titles and abstracts of 1195 studies were reviewed, and 197 of these studies were downloaded as candidate inclusion studies. Of these, 16 had suitable results for coordinate network mapping.

**Supplementary Figure 2: The robust test of sensitivity map**


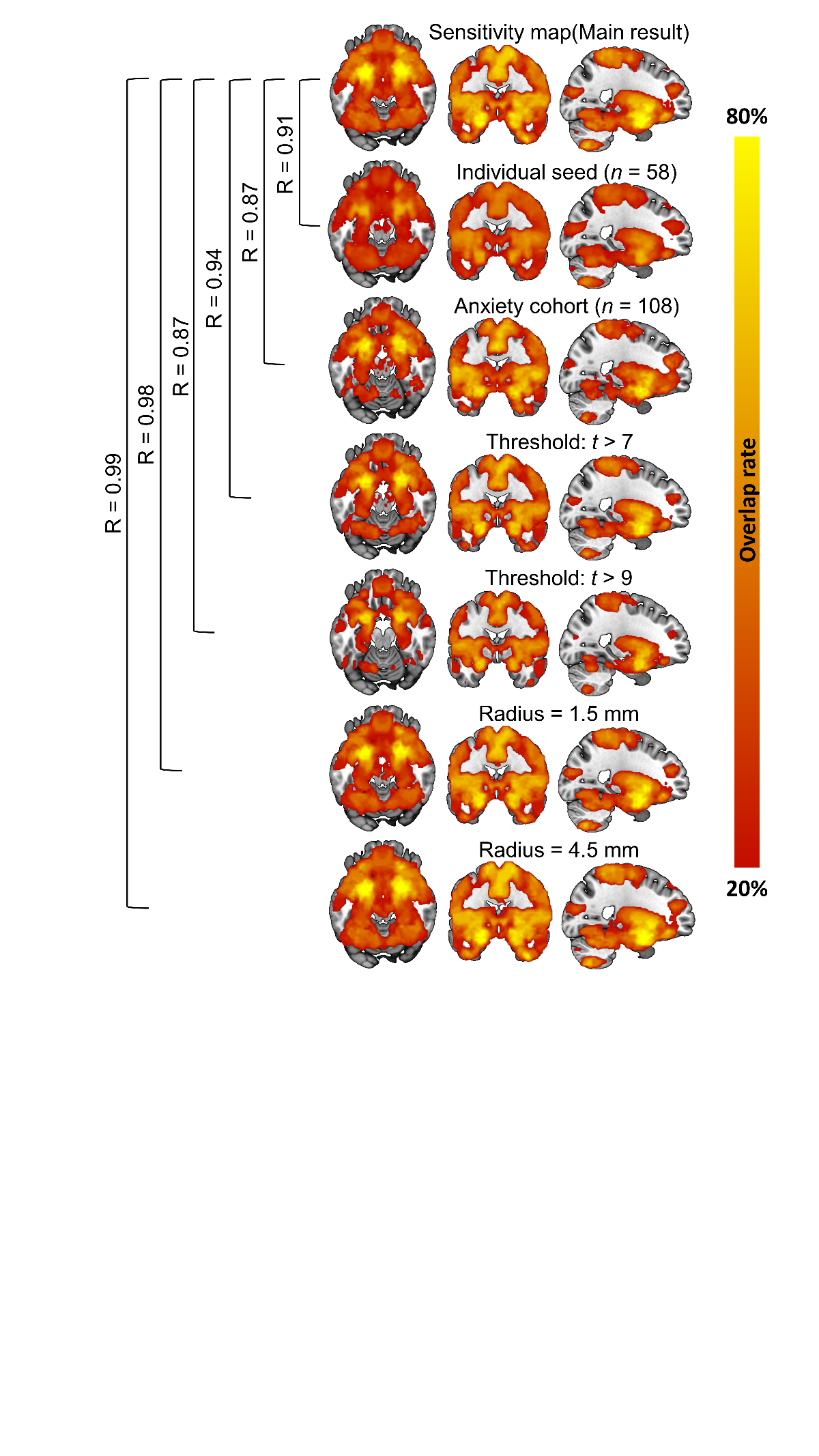


**Supplementary Figure 2:** The sensitivity map was highly reliable when repeating the original analysis with each coordinate as an individual seed (*n* = 58, spatial correlation *r* = .91), when using another independent resting-state fMRI dataset of anxiety patients (*n* = 108, *r* = .87), and when using two higher thresholds (*t* > 7, *r* = .94; *t* > 9, *r* = .87) and different seed sizes (radius 1.5 mm, *r* = .98; radius 4.5 mm, *r* = .99).

**Supplementary Figure 3: The robust test of anxiety network localization at a sensitivity map overlap threshold of 75% (12 of 16).**


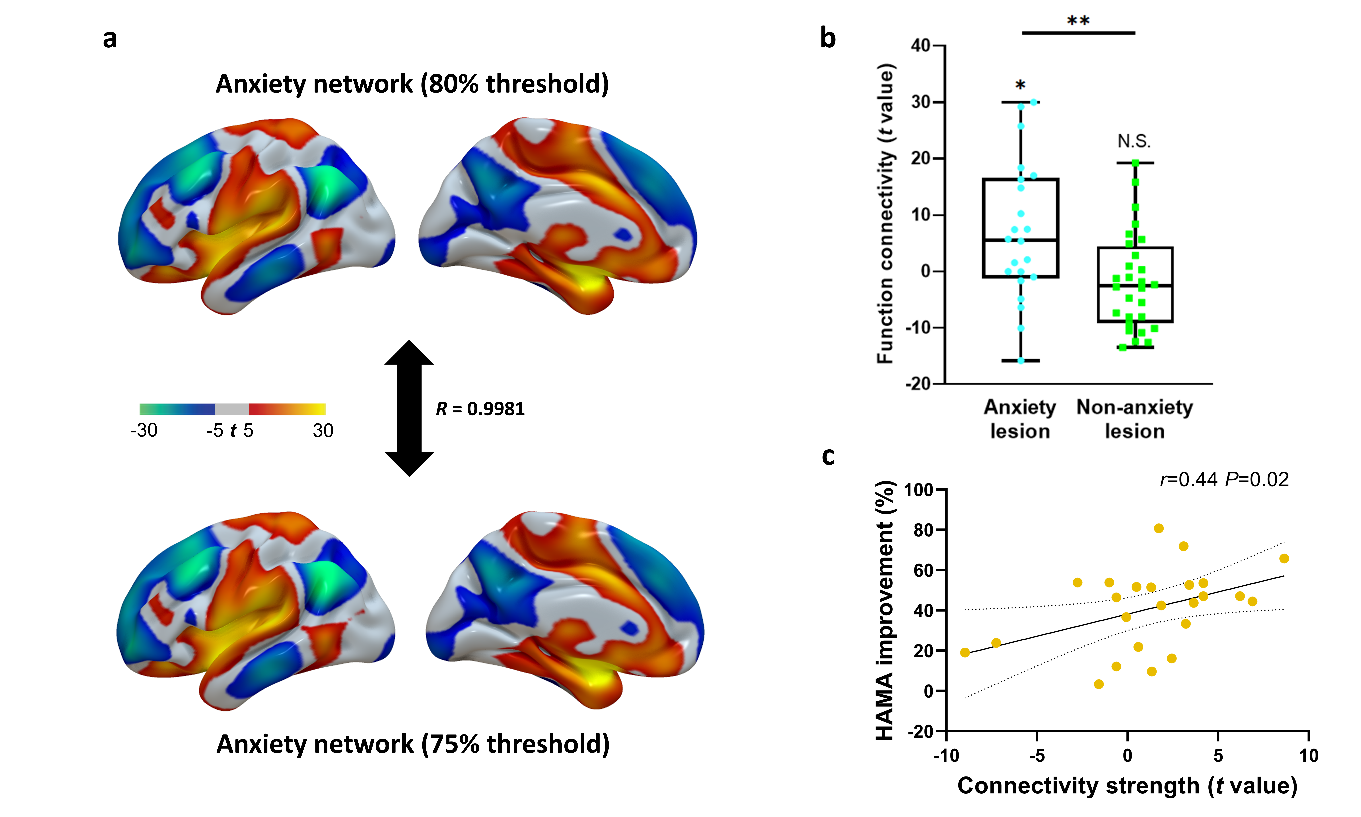


**Supplementary Figure 3:** **(a)** The anxiety network was highly reliable when regions with an overlap rate exceeding 75% (12 of 16) in the sensitivity map were used to identify the hub region (spatial correlation *r* = .9981). **(b)** Lesions in the anxiety group showed significant functional connectivity within the anxiety network (*n* = 22, *t* = 2.62, *P* = .02), while lesions in the non-anxiety group did not (*n* = 28, *t* = 1.07, *P* = .29). The average connectivity strength was also significantly higher for anxiety-associated lesions than non-anxiety-associated lesions (*t* = 2.91; *P* = .005). **(c)** the connectivity strength of rTMS targets within the anxiety network of patients with anxiety symptoms (*n* = 24) was correlated with anxiety symptom improvement (*r* = .44; *P* = .02).

**Supplementary Figure 4: Work flow for identifying anxiety-causing lesions from the literature.**


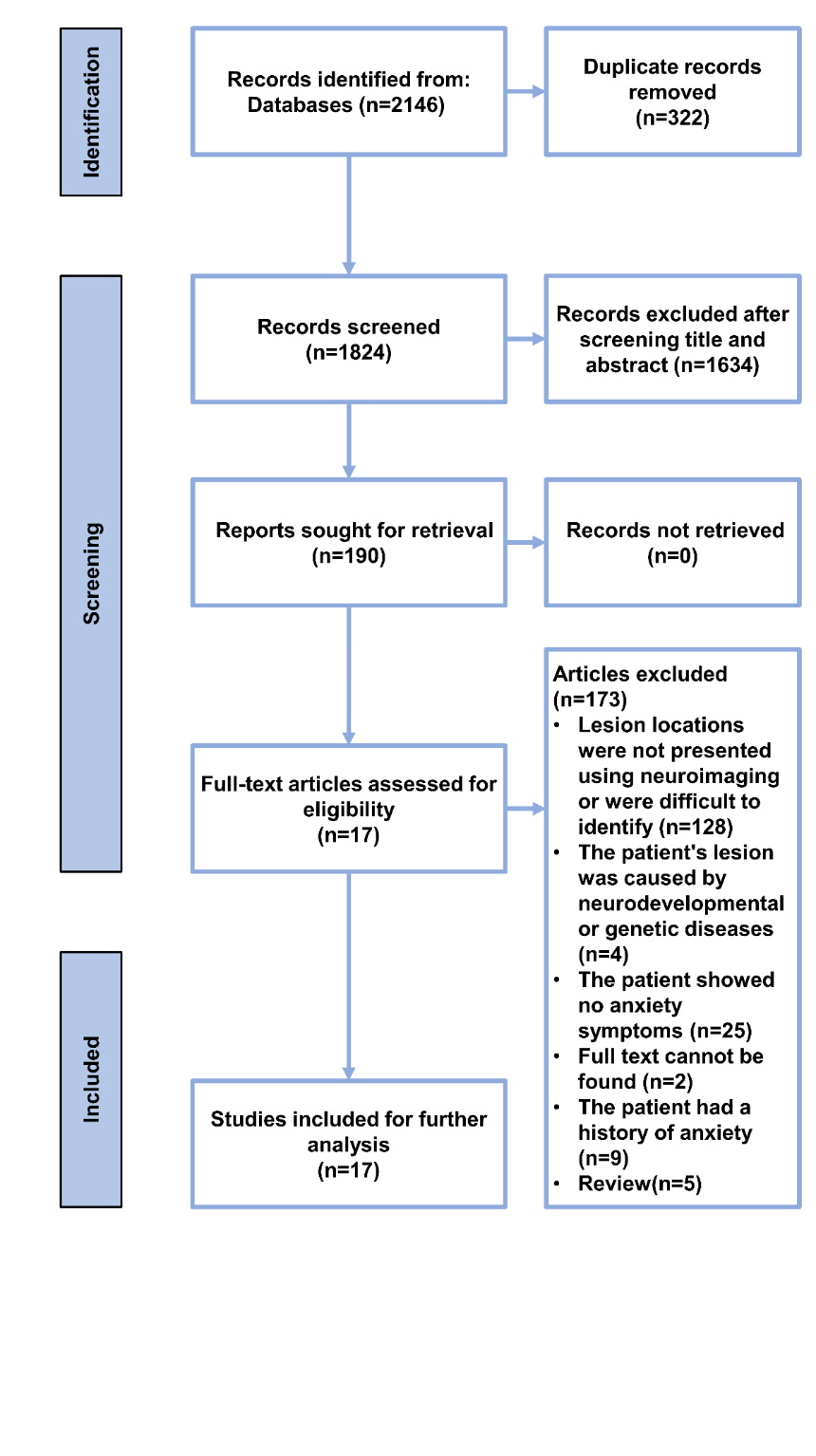


**Supplementary Figure 4:** 2146 articles matched initial search criteria. After eliminating duplicate articles, the titles and abstracts of 1824 studies were reviewed, and 190 of these studies were downloaded as candidate inclusion studies. Of these, 17 had suitable lesions for comparison with lesion that did not cause anxiety symptoms.

**Supplementary Figure 5: Another two measures of clinical outcomes (besides the improvement rate) showing significant correlations with the functional connectivity strength of target (t-value).**


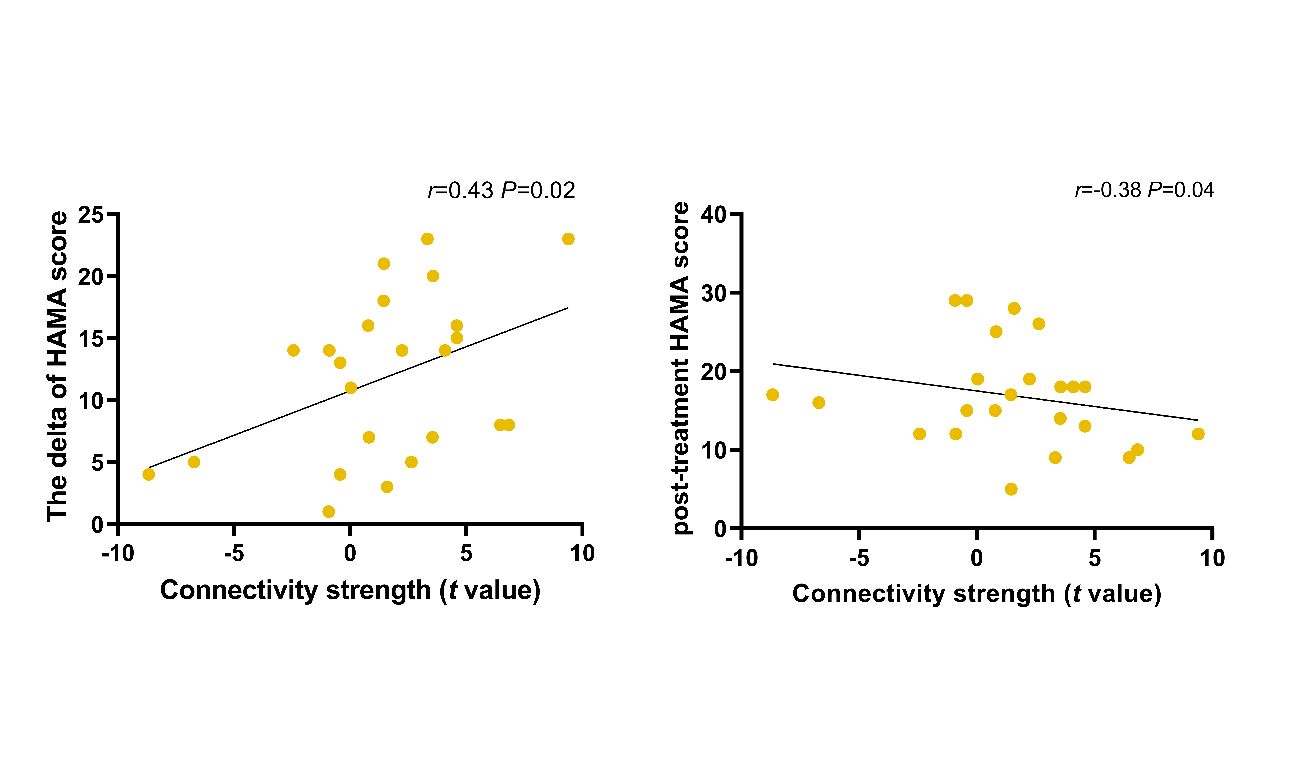


**Supplementary Figure 5:** Another two measures of clinical outcomes (besides the improvement rate) showing significant correlations with the functional connectivity strength of target (t-value).

# Reference

[1] Ashburner, J. “A fast diffeomorphic image registration algorithm”. *Neuroimage, 38*(1), 95-113. 2007.

[2] Anttila, V., Bulik-Sullivan, B., Finucane, H. K., Walters, R. K., Bras, J., Duncan, L., Escott-Price, V., Falcone, G. J., Gormley, P., Malik, R., Patsopoulos, N. A., Ripke, S., Wei, Z., Yu, D., Lee, P. H., Turley, P., Grenier-Boley, B., Chouraki, V., Kamatani, Y., Berr, C., Letenneur, L., Hannequin, D., Amouyel, P., Boland, A., Deleuze, J. F., Duron, E., Vardarajan, B. N., Reitz, C., Goate, A. M., Huentelman, M. J., Kamboh, M. I., Larson, E. B., Rogaeva, E., St George-Hyslop, P., Hakonarson, H., Kukull, W. A., Farrer, L. A., Barnes, L. L., Beach, T. G., Demirci, F. Y., Head, E., Hulette, C. M., Jicha, G. A., Kauwe, J. S. K., Kaye, J. A., Leverenz, J. B., Levey, A. I., Lieberman, A. P., Pankratz, V. S., Poon, W. W., Quinn, J. F., Saykin, A. J., Schneider, L. S., Smith, A. G., Sonnen, J. A., Stern, R. A., Van Deerlin, V. M., Van Eldik, L. J., Harold, D., Russo, G., Rubinsztein, D. C., Bayer, A., Tsolaki, M., Proitsi, P., Fox, N. C., Hampel, H., Owen, M. J., Mead, S., Passmore, P., Morgan, K., Nöthen, M. M., Rossor, M., Lupton, M. K., Hoffmann, P., Kornhuber, J., Lawlor, B., McQuillin, A., Al-Chalabi, A., Bis, J. C., Ruiz, A., Boada, M., Seshadri, S., Beiser, A., Rice, K., van der Lee, S. J., De Jager, P. L., Geschwind, D. H., Riemenschneider, M., Riedel-Heller, S., Rotter, J. I., Ransmayr, G., Hyman, B. T., Cruchaga, C., Alegret, M., Winsvold, B., Palta, P., Farh, K. H., Cuenca-Leon, E., Furlotte, N., Kurth, T., Ligthart, L., Terwindt, G. M., Freilinger, T., Ran, C., Gordon, S. D., Borck, G., Adams, H. H. H., Lehtimäki, T., Wedenoja, J., Buring, J. E., Schürks, M., Hrafnsdottir, M., Hottenga, J. J., Penninx, B., Artto, V., Kaunisto, M., Vepsäläinen, S., Martin, N. G., Montgomery, G. W., Kurki, M. I., Hämäläinen, E., Huang, H., Huang, J., Sandor, C., Webber, C., Muller-Myhsok, B., Schreiber, S., Salomaa, V., Loehrer, E., Göbel, H., Macaya, A., Pozo-Rosich, P., Hansen, T., Werge, T., Kaprio, J., Metspalu, A., Kubisch, C., Ferrari, M. D., Belin, A. C., van den Maagdenberg, A., Zwart, J. A., Boomsma, D., Eriksson, N., Olesen, J., Chasman, D. I., Nyholt, D. R., Avbersek, A., Baum, L., Berkovic, S., Bradfield, J., Buono, R. J., Catarino, C. B., Cossette, P., De Jonghe, P., Depondt, C., Dlugos, D., Ferraro, T. N., French, J., Hjalgrim, H., Jamnadas-Khoda, J., Kälviäinen, R., Kunz, W. S., Lerche, H., Leu, C., Lindhout, D., Lo, W., Lowenstein, D., McCormack, M., Møller, R. S., Molloy, A., Ng, P. W., Oliver, K., Privitera, M., Radtke, R., Ruppert, A. K., Sander, T., Schachter, S., Schankin, C., Scheffer, I., Schoch, S., Sisodiya, S. M., Smith, P., Sperling, M., Striano, P., Surges, R., Thomas, G. N., Visscher, F., Whelan, C. D., Zara, F., Heinzen, E. L., Marson, A., Becker, F., Stroink, H., Zimprich, F., Gasser, T., Gibbs, R., Heutink, P., Martinez, M., Morris, H. R., Sharma, M., Ryten, M., Mok, K. Y., Pulit, S., Bevan, S., Holliday, E., Attia, J., Battey, T., Boncoraglio, G., Thijs, V., Chen, W. M., Mitchell, B., Rothwell, P., Sharma, P., Sudlow, C., Vicente, A., Markus, H., Kourkoulis, C., Pera, J., Raffeld, M., Silliman, S., Boraska Perica, V., Thornton, L. M., Huckins, L. M., William Rayner, N., Lewis, C. M., Gratacos, M., Rybakowski, F., Keski-Rahkonen, A., Raevuori, A., Hudson, J. I., Reichborn-Kjennerud, T., Monteleone, P., Karwautz, A., Mannik, K., Baker, J. H., O'Toole, J. K., Trace, S. E., Davis, O. S. P., Helder, S. G., Ehrlich, S., Herpertz-Dahlmann, B., Danner, U. N., van Elburg, A. A., Clementi, M., Forzan, M., Docampo, E., Lissowska, J., Hauser, J., Tortorella, A., Maj, M., Gonidakis, F., Tziouvas, K., Papezova, H., Yilmaz, Z., Wagner, G., Cohen-Woods, S., Herms, S., Julià, A., Rabionet, R., Dick, D. M., Ripatti, S., Andreassen, O. A., Espeseth, T., Lundervold, A. J., Steen, V. M., Pinto, D., Scherer, S. W., Aschauer, H., Schosser, A., Alfredsson, L., Padyukov, L., Halmi, K. A., Mitchell, J., Strober, M., Bergen, A. W., Kaye, W., Szatkiewicz, J. P., Cormand, B., Ramos-Quiroga, J. A., Sánchez-Mora, C., Ribasés, M., Casas, M., Hervas, A., Arranz, M. J., Haavik, J., Zayats, T., Johansson, S., Williams, N., Dempfle, A., Rothenberger, A., Kuntsi, J., Oades, R. D., Banaschewski, T., Franke, B., Buitelaar, J. K., Arias Vasquez, A., Doyle, A. E., Reif, A., Lesch, K. P., Freitag, C., Rivero, O., Palmason, H., Romanos, M., Langley, K., Rietschel, M., Witt, S. H., Dalsgaard, S., Børglum, A. D., Waldman, I., Wilmot, B., Molly, N., Bau, C. H. D., Crosbie, J., Schachar, R., Loo, S. K., McGough, J. J., Grevet, E. H., Medland, S. E., Robinson, E., Weiss, L. A., Bacchelli, E., Bailey, A., Bal, V., Battaglia, A., Betancur, C., Bolton, P., Cantor, R., Celestino-Soper, P., Dawson, G., De Rubeis, S., Duque, F., Green, A., Klauck, S. M., Leboyer, M., Levitt, P., Maestrini, E., Mane, S., De-Luca, D. M., Parr, J., Regan, R., Reichenberg, A., Sandin, S., Vorstman, J., Wassink, T., Wijsman, E., Cook, E., Santangelo, S., Delorme, R., Rogé, B., Magalhaes, T., Arking, D., Schulze, T. G., Thompson, R. C., Strohmaier, J., Matthews, K., Melle, I., Morris, D., Blackwood, D., McIntosh, A., Bergen, S. E., Schalling, M., Jamain, S., Maaser, A., Fischer, S. B., Reinbold, C. S., Fullerton, J. M., Guzman-Parra, J., Mayoral, F., Schofield, P. R., Cichon, S., Mühleisen, T. W., Degenhardt, F., Schumacher, J., Bauer, M., Mitchell, P. B., Gershon, E. S., Rice, J., Potash, J. B., Zandi, P. P., Craddock, N., Ferrier, I. N., Alda, M., Rouleau, G. A., Turecki, G., Ophoff, R., Pato, C., Anjorin, A., Stahl, E., Leber, M., Czerski, P. M., Cruceanu, C., Jones, I. R., Posthuma, D., Andlauer, T. F. M., Forstner, A. J., Streit, F., Baune, B. T., Air, T., Sinnamon, G., Wray, N. R., MacIntyre, D. J., Porteous, D., Homuth, G., Rivera, M., Grove, J., Middeldorp, C. M., Hickie, I., Pergadia, M., Mehta, D., Smit, J. H., Jansen, R., de Geus, E., Dunn, E., Li, Q. S., Nauck, M., Schoevers, R. A., Beekman, A. T., Knowles, J. A., Viktorin, A., Arnold, P., Barr, C. L., Bedoya-Berrio, G., Bienvenu, O. J., Brentani, H., Burton, C., Camarena, B., Cappi, C., Cath, D., Cavallini, M., Cusi, D., Darrow, S., Denys, D., Derks, E. M., Dietrich, A., Fernandez, T., Figee, M., Freimer, N., Gerber, G., Grados, M., Greenberg, E., Hanna, G. L., Hartmann, A., Hirschtritt, M. E., Hoekstra, P. J., Huang, A., Huyser, C., Illmann, C., Jenike, M., Kuperman, S., Leventhal, B., Lochner, C., Lyon, G. J., Macciardi, F., Madruga-Garrido, M., Malaty, I. A., Maras, A., McGrath, L., Miguel, E. C., Mir, P., Nestadt, G., Nicolini, H., Okun, M. S., Pakstis, A., Paschou, P., Piacentini, J., Pittenger, C., Plessen, K., Ramensky, V., Ramos, E. M., Reus, V., Richter, M. A., Riddle, M. A., Robertson, M. M., Roessner, V., Rosário, M., Samuels, J. F., Sandor, P., Stein, D. J., Tsetsos, F., Van Nieuwerburgh, F., Weatherall, S., Wendland, J. R., Wolanczyk, T., Worbe, Y., Zai, G., Goes, F. S., McLaughlin, N., Nestadt, P. S., Grabe, H. J., Depienne, C., Konkashbaev, A., Lanzagorta, N., Valencia-Duarte, A., Bramon, E., Buccola, N., Cahn, W., Cairns, M., Chong, S. A., Cohen, D., Crespo-Facorro, B., Crowley, J., Davidson, M., DeLisi, L., Dinan, T., Donohoe, G., Drapeau, E., Duan, J., Haan, L., Hougaard, D., Karachanak-Yankova, S., Khrunin, A., Klovins, J., Kučinskas, V., Lee Chee Keong, J., Limborska, S., Loughland, C., Lönnqvist, J., Maher, B., Mattheisen, M., McDonald, C., Murphy, K. C., Nenadic, I., van Os, J., Pantelis, C., Pato, M., Petryshen, T., Quested, D., Roussos, P., Sanders, A. R., Schall, U., Schwab, S. G., Sim, K., So, H. C., Stögmann, E., Subramaniam, M., Toncheva, D., Waddington, J., Walters, J., Weiser, M., Cheng, W., Cloninger, R., Curtis, D., Gejman, P. V., Henskens, F., Mattingsdal, M., Oh, S. Y., Scott, R., Webb, B., Breen, G., Churchhouse, C., Bulik, C. M., Daly, M., Dichgans, M., Faraone, S. V., Guerreiro, R., Holmans, P., Kendler, K. S., Koeleman, B., Mathews, C. A., Price, A., Scharf, J., Sklar, P., Williams, J., Wood, N. W., Cotsapas, C., Palotie, A., Smoller, J. W., Sullivan, P., Rosand, J., Corvin, A., Neale, B. M., Schott, J. M., Anney, R., Elia, J., Grigoroiu-Serbanescu, M., Edenberg, H. J., and Murray, R. “Analysis of shared heritability in common disorders of the brain”. *Science, 360*(6395). 2018.

[3] Dantendorfer, K., Amering, M., Prayer, D., Maierhofer, D., Schnider, P., and Katschnig, H. “Treatment of Koro and panic attacks after stroke”. *Anxiety, 2*(1), 53-55. 1996.

[4] Hendler, T., Goshen, E., Tadmor, R., Lustig, M., Zwas, S. T., and Zohar, J. “Evidence for striatal modulation in the presence of fixed cortical injury in obsessive-compulsive disorder (OCD)”. *Eur Neuropsychopharmacol, 9*(5), 371-376. 1999.

[5] Ohta, Y., Nagano, I., Niiya, D., Fujioka, H., Kishimoto, T., Shoji, M., and Abe, K. “Nonparaneoplastic limbic encephalitis with relapsing polychondritis”. *J Neurol Sci, 220*(1-2), 85-88. 2004.

[6] Annoni, J. M., Devuyst, G., Carota, A., Bruggimann, L., and Bogousslavsky, J. “Changes in artistic style after minor posterior stroke”. *J Neurol Neurosurg Psychiatry, 76*(6), 797-803. 2005.

[7] Mancardi, M. M., Fazzini, F., Rossi, A., and Gaggero, R. “Hashimoto's encephalopathy with selective involvement of the nucleus accumbens: a case report”. *Neuropediatrics, 36*(3), 218-220. 2005.

[8] Sansing, L. H., Tأzأn, E., Ko, M. W., Baccon, J., Lynch, D. R., and Dalmau, J. “A patient with encephalitis associated with NMDA receptor antibodies”. *Nat Clin Pract Neurol, 3*(5), 291-296. 2007.

[9] Kumar, V., Chakrabarti, S., Modi, M., and Sahoo, M. “Late-onset obsessive compulsive disorder associated with possible gliomatosis cerebri”. *World J Biol Psychiatry, 10*(4 Pt 2), 636-639. 2009.

[10] Yalud, I., Alemdar, M., Tufan, A. E., Kirmizi-Alsan, E., and Kutlu, H. “Limbic encephalitis presenting with anxiety and depression: A comprehensive neuropsychological formulation”. *World Journal of Biological Psychiatry, 10*(4), 616-619. 2009.

[11] Tang, J. F., Chen, P. L., Tang, E. J., May, T. A., and Stiver, S. I. “Dexmedetomidine controls agitation and facilitates reliable, serial neurological examinations in a non-intubated patient with traumatic brain injury”. *Neurocrit Care, 15*(1), 175-181. 2011.

[12] Chaves, C., Trzesniak, C., Derenusson, G. N., Araأjo, D., Wichert-Ana, L., Machado-de-Sousa, J. P., Carlotti, C. G., Jr., Nardi, A. E., Zuardi, A. W., de, S. C. J. A., and Hallak, J. E. “Late-onset social anxiety disorder following traumatic brain injury”. *Brain Inj, 26*(6), 882-886. 2012.

[13] Nishiyori, Y., Nishida, M., Shioda, K., Suda, S., and Kato, S. “Unilateral hippocampal infarction associated with an attempted suicide: a case report”. *J Med Case Rep, 8*, 219. 2014.

[14] Henderson, T. A., and Morries, L. D. “SPECT Perfusion Imaging Demonstrates Improvement of Traumatic Brain Injury With Transcranial Near-infrared Laser Phototherapy”. *Adv Mind Body Med, 29*(4), 27-33. 2015.

[15] Juneja, M., Kaur, S., Mishra, D., and Jain, S. “Ophelia syndrome: Hodgkin lymphoma with limbic encephalitis”. *Indian Pediatr, 52*(4), 335-336. 2015.

[16] Slotwinski, K., Ejma, M., Szczepanska, A., Budrewicz, S., and Koszewicz, M. “Pure word deafness in a patient with bilateral ischemic stroke in the superior temporal gyrus (STG)”. *Neurocase, 26*(3), 121-124. 2020.

[17] Xu, W., Zhang, C., Sun, B., and Li, D. “Sustainable Effects of 8-Year Intermittent Spinal Cord Stimulation in a Patient with Thalamic Post-Stroke Pain”. *World Neurosurg, 143*, 223-227. 2020.

[18] Bodoano Sánchez, I., Fernández-Pérez, M. D., Molera Manzano, D., Gutiérrez-Rojas, L., and Romero-Fábrega, J. C. “Visual Hallucinations in a Patient With Moyamoya Disease: A Review and Case Report”. *Cogn Behav Neurol, 34*(1), 63-69. 2021.

[19] Rieke, J. D., Lamb, D. G., Lewis, G. F., Davila, M. I., Schmalfuss, I. M., Murphy, A. J., Tran, A. B., Bottari, S. A., and Williamson, J. B. “Posttraumatic Stress Disorder Subsequent to Apparent Mild Traumatic Brain Injury”. *Cogn Behav Neurol, 34*(1), 26-37. 2021.

[20] Huang, Y. Z., Edwards, M. J., Rounis, E., Bhatia, K. P., and Rothwell, J. C. “Theta burst stimulation of the human motor cortex”. *Neuron, 45*(2), 201-206. 2005.

[21] Shirota, Y., Dhaka, S., Paulus, W., and Sommer, M. “Current direction-dependent modulation of human hand motor function by intermittent theta burst stimulation (iTBS)”. *Neurosci Lett, 650*, 109-113. 2017.

[22] Nettekoven, C., Volz, L. J., Kutscha, M., Pool, E. M., Rehme, A. K., Eickhoff, S. B., Fink, G. R., and Grefkes, C. “Dose-dependent effects of theta burst rTMS on cortical excitability and resting-state connectivity of the human motor system”. *J Neurosci, 34*(20), 6849-6859. 2014.

[23] Chen, X., Ji, G. J., Zhu, C., Bai, X., Wang, L., He, K., Gao, Y., Tao, L., Yu, F., Tian, Y., and Wang, K. “Neural Correlates of Auditory Verbal Hallucinations in Schizophrenia and the Therapeutic Response to Theta-Burst Transcranial Magnetic Stimulation”. *Schizophr Bull, 45*(2), 474-483. 2019.

[24] Makovac, E., Meeten, F., Watson, D. R., Garfinkel, S. N., Critchley, H. D., and Ottaviani, C. “Neurostructural abnormalities associated with axes of emotion dysregulation in generalized anxiety”. *Neuroimage Clin, 10*, 172-181. 2016.

[25] Ma, Z., Wang, C., Hines, C. S., Lu, X., Wu, Y., Xu, H., Li, J., Wang, Q., Pang, M., Zhong, Y., and Zhang, N. “Frontoparietal network abnormalities of gray matter volume and functional connectivity in patients with generalized anxiety disorder”. *Psychiatry Res Neuroimaging, 286*, 24-30. 2019.

[26] Chen, Y., Cui, Q., Fan, Y. S., Guo, X., Tang, Q., Sheng, W., Lei, T., Li, D., Lu, F., He, Z., Yang, Y., Hu, S., Deng, J., and Chen, H. “Progressive brain structural alterations assessed via causal analysis in patients with generalized anxiety disorder”. *Neuropsychopharmacology, 45*(10), 1689-1697. 2020.

[27] Massana, G., Serra-Grabulosa, J. M., Salgado-Pineda, P., Gastó, C., Junqué, C., Massana, J., and Mercader, J. M. “Parahippocampal gray matter density in panic disorder: a voxel-based morphometric study”. *Am J Psychiatry, 160*(3), 566-568. 2003.

[28] Yoo, H. K., Kim, M. J., Kim, S. J., Sung, Y. H., Sim, M. E., Lee, Y. S., Song, S. Y., Kee, B. S., and Lyoo, I. K. “Putaminal gray matter volume decrease in panic disorder: an optimized voxel-based morphometry study”. *Eur J Neurosci, 22*(8), 2089-2094. 2005.

[29] Hayano, F., Nakamura, M., Asami, T., Uehara, K., Yoshida, T., Roppongi, T., Otsuka, T., Inoue, T., and Hirayasu, Y. “Smaller amygdala is associated with anxiety in patients with panic disorder”. *Psychiatry Clin Neurosci, 63*(3), 266-276. 2009.

[30] Lai, C. H., and Wu, Y. T. “Fronto-temporo-insula gray matter alterations of first-episode, drug-naïve and very late-onset panic disorder patients”. *J Affect Disord, 140*(3), 285-291. 2012.

[31] Na, K. S., Ham, B. J., Lee, M. S., Kim, L., Kim, Y. K., Lee, H. J., and Yoon, H. K. “Decreased gray matter volume of the medial orbitofrontal cortex in panic disorder with agoraphobia: a preliminary study”. *Prog Neuropsychopharmacol Biol Psychiatry, 45*, 195-200. 2013.

[32] Lai, C. H., and Wu, Y. T. “The gray matter alterations in major depressive disorder and panic disorder: Putative differences in the pathogenesis”. *J Affect Disord, 186*, 1-6. 2015.

[33] Wu, H., Zhong, Y., Xu, H., Ding, H., Yuan, S., Wu, Y., Liu, G., Liu, N., and Wang, C. “Glutamic Acid Decarboxylase 1 Gene Methylation and Panic Disorder Severity: Making the Connection by Brain Gray Matter Volume”. *Front Psychiatry, 13*, 853613. 2022.

[34] Liao, W., Xu, Q., Mantini, D., Ding, J., Machado-de-Sousa, J. P., Hallak, J. E., Trzesniak, C., Qiu, C., Zeng, L., Zhang, W., Crippa, J. A., Gong, Q., and Chen, H. “Altered gray matter morphometry and resting-state functional and structural connectivity in social anxiety disorder”. *Brain Res, 1388*, 167-177. 2011.

[35] Talati, A., Pantazatos, S. P., Schneier, F. R., Weissman, M. M., and Hirsch, J. “Gray matter abnormalities in social anxiety disorder: primary, replication, and specificity studies”. *Biol Psychiatry, 73*(1), 75-84. 2013.

[36] Meng, Y., Lui, S., Qiu, C., Qiu, L., Lama, S., Huang, X., Feng, Y., Zhu, C., Gong, Q., and Zhang, W. “Neuroanatomical deficits in drug-naïve adult patients with generalized social anxiety disorder: a voxel-based morphometry study”. *Psychiatry Res, 214*(1), 9-15. 2013.

[37] Zhao, Y., Chen, L., Zhang, W., Xiao, Y., Shah, C., Zhu, H., Yuan, M., Sun, H., Yue, Q., Jia, Z., Zhang, W., Kuang, W., Gong, Q., and Lui, S. “Gray Matter Abnormalities in Non-comorbid Medication-naive Patients with Major Depressive Disorder or Social Anxiety Disorder”. *EBioMedicine, 21*, 228-235. 2017.

[38] Zhang, X., Suo, X., Yang, X., Lai, H., Pan, N., He, M., Li, Q., Kuang, W., Wang, S., and Gong, Q. “Structural and functional deficits and couplings in the cortico-striato-thalamo-cerebellar circuitry in social anxiety disorder”. *Transl Psychiatry, 12*(1), 26. 2022.

[39] Rivero, F., Marrero, R. J., Olivares, T., Peñate, W., Álvarez-Pérez, Y., Bethencourt, J. M., and Fumero, A. “A Voxel-Based Morphometric Study of Gray Matter in Specific Phobia”. *Life (Basel), 13*(1). 2022.

[40] Sader, M., Williams, J. H. G., and Waiter, G. D. “A meta-analytic investigation of grey matter differences in anorexia nervosa and autism spectrum disorder”. *Eur Eat Disord Rev, 30*(5), 560-579. 2022.

[41] Lukito, S., Norman, L., Carlisi, C., Radua, J., Hart, H., Simonoff, E., and Rubia, K. “Comparative meta-analyses of brain structural and functional abnormalities during cognitive control in attention-deficit/hyperactivity disorder and autism spectrum disorder”. *Psychol Med, 50*(6), 894-919. 2020.

[42] Gong, J., Wang, J., Chen, P., Qi, Z., Luo, Z., Wang, J., Huang, L., and Wang, Y. “Large-scale network abnormality in bipolar disorder: A multimodal meta-analysis of resting-state functional and structural magnetic resonance imaging studies”. *J Affect Disord, 292*, 9-20. 2021.

[43] Picó-Pérez, M., Moreira, P. S., de Melo Ferreira, V., Radua, J., Mataix-Cols, D., Sousa, N., Soriano-Mas, C., and Morgado, P. “Modality-specific overlaps in brain structure and function in obsessive-compulsive disorder: Multimodal meta-analysis of case-control MRI studies”. *Neurosci Biobehav Rev, 112*, 83-94. 2020.

[44] Serra-Blasco, M., Radua, J., Soriano-Mas, C., Gأ٣mez-Benlloch, A., Porta-Casterأs, D., Carulla-Roig, M., Albajes-Eizagirre, A., Arnone, D., Klauser, P., Canales-Rodrأguez, E. J., Hilbert, K., Wise, T., Cheng, Y., Kandilarova, S., Mataix-Cols, D., Vieta, E., Via, E., and Cardoner, N. “Structural brain correlates in major depression, anxiety disorders and post-traumatic stress disorder: A voxel-based morphometry meta-analysis”. *Neurosci Biobehav Rev, 129*, 269-281. 2021.

[45] Wan, X., Zhang, S., Wang, W., Su, X., Li, J., Yang, X., Tan, Q., Yue, Q., and Gong, Q. “Gray matter abnormalities in Tourette Syndrome: a meta-analysis of voxel-based morphometry studies”. *Transl Psychiatry, 11*(1), 287. 2021.

[46] Castro-Fornieles, J., Bargalló, N., Lázaro, L., Andrés, S., Falcon, C., Plana, M. T., and Junqué, C. “A cross-sectional and follow-up voxel-based morphometric MRI study in adolescent anorexia nervosa”. *J Psychiatr Res, 43*(3), 331-340. 2009.

[47] Suchan, B., Busch, M., Schulte, D., Grönemeyer, D., Herpertz, S., and Vocks, S. “Reduction of gray matter density in the extrastriate body area in women with anorexia nervosa”. *Behav Brain Res, 206*(1), 63-67. 2010.

[48] Boghi, A., Sterpone, S., Sales, S., D'Agata, F., Bradac, G. B., Zullo, G., and Munno, D. “In vivo evidence of global and focal brain alterations in anorexia nervosa”. *Psychiatry Res, 192*(3), 154-159. 2011.

[49] Brooks, S. J., Barker, G. J., O'Daly, O. G., Brammer, M., Williams, S. C., Benedict, C., Schiöth, H. B., Treasure, J., and Campbell, I. C. “Restraint of appetite and reduced regional brain volumes in anorexia nervosa: a voxel-based morphometric study”. *Bmc Psychiatry, 11*, 179. 2011.

[50] Gaudio, S., Nocchi, F., Franchin, T., Genovese, E., Cannatà, V., Longo, D., and Fariello, G. “Gray matter decrease distribution in the early stages of Anorexia Nervosa restrictive type in adolescents”. *Psychiatry Res, 191*(1), 24-30. 2011.

[51] Joos, A., Hartmann, A., Glauche, V., Perlov, E., Unterbrink, T., Saum, B., Tüscher, O., Tebartz van Elst, L., and Zeeck, A. “Grey matter deficit in long-term recovered anorexia nervosa patients”. *Eur Eat Disord Rev, 19*(1), 59-63. 2011.

[52] Friederich, H. C., Walther, S., Bendszus, M., Biller, A., Thomann, P., Zeigermann, S., Katus, T., Brunner, R., Zastrow, A., and Herzog, W. “Grey matter abnormalities within cortico-limbic-striatal circuits in acute and weight-restored anorexia nervosa patients”. *Neuroimage, 59*(2), 1106-1113. 2012.

[53] Fonville, L., Giampietro, V., Williams, S. C., Simmons, A., and Tchanturia, K. “Alterations in brain structure in adults with anorexia nervosa and the impact of illness duration”. *Psychol Med, 44*(9), 1965-1975. 2014.

[54] Bär, K. J., de la Cruz, F., Berger, S., Schultz, C. C., and Wagner, G. “Structural and functional differences in the cingulate cortex relate to disease severity in anorexia nervosa”. *J Psychiatry Neurosci, 40*(4), 269-279. 2015.

[55] D'Agata, F., Caroppo, P., Amianto, F., Spalatro, A., Caglio, M. M., Bergui, M., Lavagnino, L., Righi, D., Abbate-Daga, G., Pinessi, L., Mortara, P., and Fassino, S. “Brain correlates of alexithymia in eating disorders: A voxel-based morphometry study”. *Psychiatry Clin Neurosci, 69*(11), 708-716. 2015.

[56] Fujisawa, T. X., Yatsuga, C., Mabe, H., Yamada, E., Masuda, M., and Tomoda, A. “Anorexia Nervosa during Adolescence Is Associated with Decreased Gray Matter Volume in the Inferior Frontal Gyrus”. *PLoS One, 10*(6), e0128548. 2015.

[57] Seitz, J., Walter, M., Mainz, V., Herpertz-Dahlmann, B., Konrad, K., and von Polier, G. “Brain volume reduction predicts weight development in adolescent patients with anorexia nervosa”. *J Psychiatr Res, 68*, 228-237. 2015.

[58] van Opstal, A. M., Westerink, A. M., Teeuwisse, W. M., van der Geest, M. A., van Furth, E. F., and van der Grond, J. “Hypothalamic BOLD response to glucose intake and hypothalamic volume are similar in anorexia nervosa and healthy control subjects”. *Front Neurosci, 9*, 159. 2015.

[59] Kohmura, K., Adachi, Y., Tanaka, S., Katayama, H., Imaeda, M., Kawano, N., Nishioka, K., Ando, M., Iidaka, T., and Ozaki, N. “Regional decrease in gray matter volume is related to body dissatisfaction in anorexia nervosa”. *Psychiatry Res Neuroimaging, 267*, 51-58. 2017.

[60] Martin Monzon, B., Henderson, L. A., Madden, S., Macefield, V. G., Touyz, S., Kohn, M. R., Clarke, S., Foroughi, N., and Hay, P. “Grey matter volume in adolescents with anorexia nervosa and associated eating disorder symptoms”. *Eur J Neurosci, 46*(7), 2297-2307. 2017.

[61] Nickel, K., Joos, A., Tebartz van Elst, L., Matthis, J., Holovics, L., Endres, D., Zeeck, A., Hartmann, A., Tüscher, O., and Maier, S. “Recovery of cortical volume and thickness after remission from acute anorexia nervosa”. *Int J Eat Disord, 51*(9), 1056-1069. 2018.

[62] Boddaert, N., Chabane, N., Gervais, H., Good, C. D., Bourgeois, M., Plumet, M. H., Barthélémy, C., Mouren, M. C., Artiges, E., Samson, Y., Brunelle, F., Frackowiak, R. S., and Zilbovicius, M. “Superior temporal sulcus anatomical abnormalities in childhood autism: a voxel-based morphometry MRI study”. *Neuroimage, 23*(1), 364-369. 2004.

[63] McAlonan, G. M., Suckling, J., Wong, N., Cheung, V., Lienenkaemper, N., Cheung, C., and Chua, S. E. “Distinct patterns of grey matter abnormality in high-functioning autism and Asperger's syndrome”. *J Child Psychol Psychiatry, 49*(12), 1287-1295. 2008.

[64] Kosaka, H., Omori, M., Munesue, T., Ishitobi, M., Matsumura, Y., Takahashi, T., Narita, K., Murata, T., Saito, D. N., Uchiyama, H., Morita, T., Kikuchi, M., Mizukami, K., Okazawa, H., Sadato, N., and Wada, Y. “Smaller insula and inferior frontal volumes in young adults with pervasive developmental disorders”. *Neuroimage, 50*(4), 1357-1363. 2010.

[65] Cheng, Y., Chou, K. H., Fan, Y. T., and Lin, C. P. “ANS: aberrant neurodevelopment of the social cognition network in adolescents with autism spectrum disorders”. *PLoS One, 6*(4), e18905. 2011.

[66] Hyde, K. L., Samson, F., Evans, A. C., and Mottron, L. “Neuroanatomical differences in brain areas implicated in perceptual and other core features of autism revealed by cortical thickness analysis and voxel-based morphometry”. *Hum Brain Mapp, 31*(4), 556-566. 2010.

[67] Kurth, F., Narr, K. L., Woods, R. P., O'Neill, J., Alger, J. R., Caplan, R., McCracken, J. T., Toga, A. W., and Levitt, J. G. “Diminished gray matter within the hypothalamus in autism disorder: a potential link to hormonal effects?”. *Biol Psychiatry, 70*(3), 278-282. 2011.

[68] Riva, D., Annunziata, S., Contarino, V., Erbetta, A., Aquino, D., and Bulgheroni, S. “Gray matter reduction in the vermis and CRUS-II is associated with social and interaction deficits in low-functioning children with autistic spectrum disorders: a VBM-DARTEL Study”. *Cerebellum, 12*(5), 676-685. 2013.

[69] Sato, W., Kochiyama, T., Uono, S., Yoshimura, S., Kubota, Y., Sawada, R., Sakihama, M., and Toichi, M. “Reduced Gray Matter Volume in the Social Brain Network in Adults with Autism Spectrum Disorder”. *Front Hum Neurosci, 11*, 395. 2017.

[70] Ni, H. C., Lin, H. Y., Tseng, W. I., Chiu, Y. N., Wu, Y. Y., Tsai, W. C., and Gau, S. S. “Neural correlates of impaired self-regulation in male youths with autism spectrum disorder: A voxel-based morphometry study”. *Prog Neuropsychopharmacol Biol Psychiatry, 82*, 233-241. 2018.

[71] Yang, Q., Huang, P., Li, C., Fang, P., Zhao, N., Nan, J., Wang, B., Gao, W., and Cui, L. B. “Mapping alterations of gray matter volume and white matter integrity in children with autism spectrum disorder: evidence from fMRI findings”. *Neuroreport, 29*(14), 1188-1192. 2018.

[72] Lochhead, R. A., Parsey, R. V., Oquendo, M. A., and Mann, J. J. “Regional brain gray matter volume differences in patients with bipolar disorder as assessed by optimized voxel-based morphometry”. *Biol Psychiatry, 55*(12), 1154-1162. 2004.

[73] Lyoo, I. K., Kim, M. J., Stoll, A. L., Demopulos, C. M., Parow, A. M., Dager, S. R., Friedman, S. D., Dunner, D. L., and Renshaw, P. F. “Frontal lobe gray matter density decreases in bipolar I disorder”. *Biol Psychiatry, 55*(6), 648-651. 2004.

[74] Nugent, A. C., Milham, M. P., Bain, E. E., Mah, L., Cannon, D. M., Marrett, S., Zarate, C. A., Pine, D. S., Price, J. L., and Drevets, W. C. “Cortical abnormalities in bipolar disorder investigated with MRI and voxel-based morphometry”. *Neuroimage, 30*(2), 485-497. 2006.

[75] Chen, X., Wen, W., Malhi, G. S., Ivanovski, B., and Sachdev, P. S. “Regional gray matter changes in bipolar disorder: a voxel-based morphometric study”. *Aust N Z J Psychiatry, 41*(4), 327-336. 2007.

[76] Haldane, M., Cunningham, G., Androutsos, C., and Frangou, S. “Structural brain correlates of response inhibition in Bipolar Disorder I”. *J Psychopharmacol, 22*(2), 138-143. 2008.

[77] Almeida, J. R., Akkal, D., Hassel, S., Travis, M. J., Banihashemi, L., Kerr, N., Kupfer, D. J., and Phillips, M. L. “Reduced gray matter volume in ventral prefrontal cortex but not amygdala in bipolar disorder: significant effects of gender and trait anxiety”. *Psychiatry Res, 171*(1), 54-68. 2009.

[78] Ha, T. H., Ha, K., Kim, J. H., and Choi, J. E. “Regional brain gray matter abnormalities in patients with bipolar II disorder: a comparison study with bipolar I patients and healthy controls”. *Neurosci Lett, 456*(1), 44-48. 2009.

[79] Stanfield, A. C., Moorhead, T. W., Job, D. E., McKirdy, J., Sussmann, J. E., Hall, J., Giles, S., Johnstone, E. C., Lawrie, S. M., and McIntosh, A. M. “Structural abnormalities of ventrolateral and orbitofrontal cortex in patients with familial bipolar disorder”. *Bipolar Disord, 11*(2), 135-144. 2009.

[80] Tost, H., Ruf, M., Schmäl, C., Schulze, T. G., Knorr, C., Vollmert, C., Bösshenz, K., Ende, G., Meyer-Lindenberg, A., Henn, F. A., and Rietschel, M. “Prefrontal-temporal gray matter deficits in bipolar disorder patients with persecutory delusions”. *J Affect Disord, 120*(1-3), 54-61. 2010.

[81] Brown, G. G., Lee, J. S., Strigo, I. A., Caligiuri, M. P., Meloy, M. J., and Lohr, J. “Voxel-based morphometry of patients with schizophrenia or bipolar I disorder: a matched control study”. *Psychiatry Res, 194*(2), 149-156. 2011.

[82] Frangou, S. “Brain structural and functional correlates of resilience to Bipolar Disorder”. *Front Hum Neurosci, 5*, 184. 2011.

[83] Hajek, T., Cullis, J., Novak, T., Kopecek, M., Höschl, C., Blagdon, R., O'Donovan, C., Bauer, M., Young, L. T., Macqueen, G., and Alda, M. “Hippocampal volumes in bipolar disorders: opposing effects of illness burden and lithium treatment”. *Bipolar Disord, 14*(3), 261-270. 2012.

[84] Ambrosi, E., Rossi-Espagnet, M. C., Kotzalidis, G. D., Comparelli, A., Del Casale, A., Carducci, F., Romano, A., Manfredi, G., Tatarelli, R., Bozzao, A., and Girardi, P. “Structural brain alterations in bipolar disorder II: a combined voxel-based morphometry (VBM) and diffusion tensor imaging (DTI) study”. *J Affect Disord, 150*(2), 610-615. 2013.

[85] Redlich, R., Almeida, J. J., Grotegerd, D., Opel, N., Kugel, H., Heindel, W., Arolt, V., Phillips, M. L., and Dannlowski, U. “Brain morphometric biomarkers distinguishing unipolar and bipolar depression. A voxel-based morphometry-pattern classification approach”. *JAMA Psychiatry, 71*(11), 1222-1230. 2014.

[86] Sarıçiçek, A., Yalın, N., Hıdıroğlu, C., Çavuşoğlu, B., Taş, C., Ceylan, D., Zorlu, N., Ada, E., Tunca, Z., and Özerdem, A. “Neuroanatomical correlates of genetic risk for bipolar disorder: A voxel-based morphometry study in bipolar type I patients and healthy first degree relatives”. *J Affect Disord, 186*, 110-118. 2015.

[87] Shepherd, A. M., Quidé, Y., Laurens, K. R., O'Reilly, N., Rowland, J. E., Mitchell, P. B., Carr, V. J., and Green, M. J. “Shared intermediate phenotypes for schizophrenia and bipolar disorder: neuroanatomical features of subtypes distinguished by executive dysfunction”. *J Psychiatry Neurosci, 40*(1), 58-68. 2015.

[88] Alonso-Lana, S., Goikolea, J. M., Bonnin, C. M., Sarró, S., Segura, B., Amann, B. L., Monté, G. C., Moro, N., Fernandez-Corcuera, P., Maristany, T., Salvador, R., Vieta, E., Pomarol-Clotet, E., and McKenna, P. J. “Structural and Functional Brain Correlates of Cognitive Impairment in Euthymic Patients with Bipolar Disorder”. *PLoS One, 11*(7), e0158867. 2016.

[89] Matsubara, T., Matsuo, K., Harada, K., Nakano, M., Nakashima, M., Watanuki, T., Egashira, K., Furukawa, M., Matsunaga, N., and Watanabe, Y. “Distinct and Shared Endophenotypes of Neural Substrates in Bipolar and Major Depressive Disorders”. *PLoS One, 11*(12), e0168493. 2016.

[90] Poletti, S., Vai, B., Smeraldi, E., Cavallaro, R., Colombo, C., and Benedetti, F. “Adverse childhood experiences influence the detrimental effect of bipolar disorder and schizophrenia on cortico-limbic grey matter volumes”. *J Affect Disord, 189*, 290-297. 2016.

[91] Sani, G., Chiapponi, C., Piras, F., Ambrosi, E., Simonetti, A., Danese, E., Janiri, D., Brugnoli, R., De Filippis, S., Caltagirone, C., Girardi, P., and Spalletta, G. “Gray and white matter trajectories in patients with bipolar disorder”. *Bipolar Disord, 18*(1), 52-62. 2016.

[92] Altamura, A. C., Maggioni, E., Dhanoa, T., Ciappolino, V., Paoli, R. A., Cremaschi, L., Prunas, C., Orsenigo, G., Caletti, E., Cinnante, C. M., Triulzi, F. M., Dell'Osso, B., Yatham, L., and Brambilla, P. “The impact of psychosis on brain anatomy in bipolar disorder: A structural MRI study”. *J Affect Disord, 233*, 100-109. 2018.

[93] Lee, J., Choi, S., Kang, J., Won, E., Tae, W. S., Lee, M. S., and Ham, B. J. “Structural characteristics of the brain reward circuit regions in patients with bipolar I disorder: A voxel-based morphometric study”. *Psychiatry Res Neuroimaging, 269*, 82-89. 2017.

[94] Maggioni, E., Crespo-Facorro, B., Nenadic, I., Benedetti, F., Gaser, C., Sauer, H., Roiz-Santiañez, R., Poletti, S., Marinelli, V., Bellani, M., Perlini, C., Ruggeri, M., Altamura, A. C., Diwadkar, V. A., and Brambilla, P. “Common and distinct structural features of schizophrenia and bipolar disorder: The European Network on Psychosis, Affective disorders and Cognitive Trajectory (ENPACT) study”. *PLoS One, 12*(11), e0188000. 2017.

[95] Wang, X., Zhao, N., Shi, J., Wu, Y., Liu, J., Xiao, Q., and Hu, J. “Discussion on Patients with Bipolar Disorder and Depressive Episode by Ratio Low Frequency Amplitude Combined with Grey Matter Volume Analysis”. *J Med Syst, 43*(5), 117. 2019.

[96] Lee, D. K., Lee, H., Park, K., Joh, E., Kim, C. E., and Ryu, S. “Common gray and white matter abnormalities in schizophrenia and bipolar disorder”. *PLoS One, 15*(5), e0232826. 2020.

[97] Li, H., Cui, L., Cao, L., Zhang, Y., Liu, Y., Deng, W., and Zhou, W. “Identification of bipolar disorder using a combination of multimodality magnetic resonance imaging and machine learning techniques”. *Bmc Psychiatry, 20*(1), 488. 2020.

[98] Song, H., Chon, M. W., Ryu, V., Yu, R., Lee, D. K., Lee, H., Lee, W., Lee, J. H., and Park, D. Y. “Cortical Volumetric Correlates of Childhood Trauma, Anxiety, and Impulsivity in Bipolar Disorder”. *Psychiatry Investig, 17*(7), 627-635. 2020.

[99] Vai, B., Parenti, L., Bollettini, I., Cara, C., Verga, C., Melloni, E., Mazza, E., Poletti, S., Colombo, C., and Benedetti, F. “Predicting differential diagnosis between bipolar and unipolar depression with multiple kernel learning on multimodal structural neuroimaging”. *Eur Neuropsychopharmacol, 34*, 28-38. 2020.

[100] Pujol, J., Soriano-Mas, C., Alonso, P., Cardoner, N., Menchón, J. M., Deus, J., and Vallejo, J. “Mapping structural brain alterations in obsessive-compulsive disorder”. *Arch Gen Psychiatry, 61*(7), 720-730. 2004.

[101] Valente, A. A., Jr., Miguel, E. C., Castro, C. C., Amaro, E., Jr., Duran, F. L., Buchpiguel, C. A., Chitnis, X., McGuire, P. K., and Busatto, G. F. “Regional gray matter abnormalities in obsessive-compulsive disorder: a voxel-based morphometry study”. *Biol Psychiatry, 58*(6), 479-487. 2005.

[102] Gilbert, A. R., Mataix-Cols, D., Almeida, J. R., Lawrence, N., Nutche, J., Diwadkar, V., Keshavan, M. S., and Phillips, M. L. “Brain structure and symptom dimension relationships in obsessive-compulsive disorder: a voxel-based morphometry study”. *J Affect Disord, 109*(1-2), 117-126. 2008.

[103] Koprivovأ, J., Horأcek, J., Tintera, J., Prasko, J., Raszka, M., Ibrahim, I., and Hأ٦schl, C. “Medial frontal and dorsal cortical morphometric abnormalities are related to obsessive-compulsive disorder”. *Neurosci Lett, 464*(1), 62-66. 2009.

[104] Togao, O., Yoshiura, T., Nakao, T., Nabeyama, M., Sanematsu, H., Nakagawa, A., Noguchi, T., Hiwatashi, A., Yamashita, K., Nagao, E., Kanba, S., and Honda, H. “Regional gray and white matter volume abnormalities in obsessive-compulsive disorder: a voxel-based morphometry study”. *Psychiatry Res, 184*(1), 29-37. 2010.

[105] Hou, J., Song, L., Zhang, W., Wu, W., Wang, J., Zhou, D., Qu, W., Guo, J., Gu, S., He, M., Xie, B., and Li, H. “Morphologic and functional connectivity alterations of corticostriatal and default mode network in treatment-naأve patients with obsessive-compulsive disorder”. *PLoS One, 8*(12), e83931. 2013.

[106] Subirأ, M., Alonso, P., Segalأs, C., Real, E., Lأ٣pez-Solأ, C., Pujol, J., Martأnez-Zalacaأn, I., Harrison, B. J., Menchأ٣n, J. M., Cardoner, N., and Soriano-Mas, C. “Brain structural alterations in obsessive-compulsive disorder patients with autogenous and reactive obsessions”. *PLoS One, 8*(9), e75273. 2013.

[107] Hashimoto, N., Nakaaki, S., Kawaguchi, A., Sato, J., Kasai, H., Nakamae, T., Narumoto, J., Miyata, J., Furukawa, T. A., and Mimura, M. “Brain structural abnormalities in behavior therapy-resistant obsessive-compulsive disorder revealed by voxel-based morphometry”. *Neuropsychiatr Dis Treat, 10*, 1987-1996. 2014.

[108] Tang, W., Huang, X., Li, B., Jiang, X., Li, F., Xu, J., Yang, Y., and Gong, Q. “Structural brain abnormalities correlate with clinical features in patients with drug-naأve OCD: A DARTEL-enhanced voxel-based morphometry study”. *Behav Brain Res, 294*, 72-80. 2015.

[109] Tang, W., Zhu, Q., Gong, X., Zhu, C., Wang, Y., and Chen, S. “Cortico-striato-thalamo-cortical circuit abnormalities in obsessive-compulsive disorder: A voxel-based morphometric and fMRI study of the whole brain”. *Behav Brain Res, 313*, 17-22. 2016.

[110] Moreira, P. S., Marques, P., Soriano-Mas, C., Magalhأes, R., Sousa, N., Soares, J. M., and Morgado, P. “The neural correlates of obsessive-compulsive disorder: a multimodal perspective”. *Transl Psychiatry, 7*(8), e1224. 2017.

[111] Moon, C. M., and Jeong, G. W. “Associations of neurofunctional, morphometric and metabolic abnormalities with clinical symptom severity and recognition deficit in obsessive-compulsive disorder”. *J Affect Disord, 227*, 603-612. 2018.

[112] Corbo, V., Clأment, M. H., Armony, J. L., Pruessner, J. C., and Brunet, A. “Size versus shape differences: contrasting voxel-based and volumetric analyses of the anterior cingulate cortex in individuals with acute posttraumatic stress disorder”. *Biol Psychiatry, 58*(2), 119-124. 2005.

[113] Chen, S., Xia, W., Li, L., Liu, J., He, Z., Zhang, Z., Yan, L., Zhang, J., and Hu, D. “Gray matter density reduction in the insula in fire survivors with posttraumatic stress disorder: a voxel-based morphometric study”. *Psychiatry Res, 146*(1), 65-72. 2006.

[114] Li, L., Chen, S., Liu, J., Zhang, J., He, Z., and Lin, X. “Magnetic resonance imaging and magnetic resonance spectroscopy study of deficits in hippocampal structure in fire victims with recent-onset posttraumatic stress disorder”. *Can J Psychiatry, 51*(7), 431-437. 2006.

[115] Bryant, R. A., Felmingham, K., Whitford, T. J., Kemp, A., Hughes, G., Peduto, A., and Williams, L. M. “Rostral anterior cingulate volume predicts treatment response to cognitive-behavioural therapy for posttraumatic stress disorder”. *J Psychiatry Neurosci, 33*(2), 142-146. 2008.

[116] Felmingham, K., Williams, L. M., Whitford, T. J., Falconer, E., Kemp, A. H., Peduto, A., and Bryant, R. A. “Duration of posttraumatic stress disorder predicts hippocampal grey matter loss”. *Neuroreport, 20*(16), 1402-1406. 2009.

[117] Sui, S.-G., Zhang, Y., Wu, M.-X., Xu, J.-M., Duan, L., Weng, X.-C., Shan, B.-C., and Li, L.-J. “Abnormal cerebellum density in victims of rape with post-traumatic stress disorder: Voxel-based analysis of magnetic resonance imaging investigation”. *Asia-Pacific Psychiatry, 2*(3), 129-135. 2010.

[118] Sui, S. G., Wu, M. X., King, M. E., Zhang, Y., Ling, L., Xu, J. M., Weng, X. C., Duan, L., Shan, B. C., and Li, L. J. “Abnormal grey matter in victims of rape with PTSD in Mainland China: a voxel-based morphometry study”. *Acta Neuropsychiatr, 22*(3), 118-126. 2010.

[119] Zhang, J., Tan, Q., Yin, H., Zhang, X., Huan, Y., Tang, L., Wang, H., Xu, J., and Li, L. “Decreased gray matter volume in the left hippocampus and bilateral calcarine cortex in coal mine flood disaster survivors with recent onset PTSD”. *Psychiatry Res, 192*(2), 84-90. 2011.

[120] Nardo, D., Hأ٦gberg, G., Lanius, R. A., Jacobsson, H., Jonsson, C., Hأllstrأ٦m, T., and Pagani, M. “Gray matter volume alterations related to trait dissociation in PTSD and traumatized controls”. *Acta Psychiatr Scand, 128*(3), 222-233. 2013.

[121] Rocha-Rego, V., Pereira, M. G., Oliveira, L., Mendlowicz, M. V., Fiszman, A., Marques-Portella, C., Berger, W., Chu, C., Joffily, M., Moll, J., Mari, J. J., Figueira, I., and Volchan, E. “Decreased premotor cortex volume in victims of urban violence with posttraumatic stress disorder”. *PLoS One, 7*(8), e42560. 2012.

[122] Tavanti, M., Battaglini, M., Borgogni, F., Bossini, L., Calossi, S., Marino, D., Vatti, G., Pieraccini, F., Federico, A., Castrogiovanni, P., and De Stefano, N. “Evidence of diffuse damage in frontal and occipital cortex in the brain of patients with post-traumatic stress disorder”. *Neurol Sci, 33*(1), 59-68. 2012.

[123] Tan, L., Zhang, L., Qi, R., Lu, G., Li, L., Liu, J., and Li, W. “Brain structure in post-traumatic stress disorder: A voxel-based morphometry analysis”. *Neural Regen Res, 8*(26), 2405-2414. 2013.

[124] Cheng, B., Huang, X., Li, S., Hu, X., Luo, Y., Wang, X., Yang, X., Qiu, C., Yang, Y., Zhang, W., Bi, F., Roberts, N., and Gong, Q. “Gray Matter Alterations in Post-Traumatic Stress Disorder, Obsessive-Compulsive Disorder, and Social Anxiety Disorder”. *Front Behav Neurosci, 9*, 219. 2015.

[125] Bossini, L., Santarnecchi, E., Casolaro, I., Koukouna, D., Caterini, C., Cecchini, F., Fortini, V., Vatti, G., Marino, D., Fernandez, I., Rossi, A., and Fagiolini, A. “Morphovolumetric changes after EMDR treatment in drug-naأve PTSD patients”. *Riv Psichiatr, 52*(1), 24-31. 2017.

[126] O'Doherty, D. C. M., Tickell, A., Ryder, W., Chan, C., Hermens, D. F., Bennett, M. R., and Lagopoulos, J. “Frontal and subcortical grey matter reductions in PTSD”. *Psychiatry Res Neuroimaging, 266*, 1-9. 2017.

[127] Wittfoth, M., Bornmann, S., Peschel, T., Grosskreutz, J., Glahn, A., Buddensiek, N., Becker, H., Dengler, R., and Mأller-Vahl, K. R. “Lateral frontal cortex volume reduction in Tourette syndrome revealed by VBM”. *BMC Neurosci, 13*, 17. 2012.
